# Meta-analysis of the effect of plyometric training on the athletic performance of youth basketball players

**DOI:** 10.3389/fphys.2024.1427291

**Published:** 2024-09-20

**Authors:** Jing-Yi Zhou, Xin Wang, Lei Hao, Xiao-Wen Ran, Wei Wei

**Affiliations:** ^1^ College of Physical Education and Sport, Beijing Normal University, Beijing, China; ^2^ Hebei Oriental University, School of Humanities, Langfang, China; ^3^ College of Physical Education, Shenyang Sport University, Shenyang, China; ^4^ Department of Ultrasound, Second Hospital of Shanxi Medical University, Taiyuan, China

**Keywords:** plyometric, youth, basketball, sports performance, meta analysis

## Abstract

**Objective:**

To investigate the effects of Plyometric Training (PT) on the athletic performance of youth basketball players (age 5–17.99) and to provide a theoretical basis for applying PT in basketball training practice.

**Method:**

PubMed, Web of Science, Cochrane Library, EBSCO and other data platforms were searched, and Meta-analysis was performed using STATA 16.0 software.

**Result:**

A total of 24 studies were included, with a sample size of 738 participants. The results showed that PT improved jumping, linear sprinting, change of direction (COD) speed, and balance in youth basketball players (*p* < 0.05) but did not significantly improve lower limb strength (*p* > 0.05). The results of subgroup analyses showed that:1) Regarding the effect of PT on different aspects of athletic performance, enhancements were found for vertical jump, 5–10 m, 20–30 m sprinting ability, velocity-oriented and force-oriented COD speed, and dynamic balance ability of youth basketball players. 2) When analyzing different participant subgroups, basketball players aged 5 to 10.99 and 11–14.99 years appeared to improve their jump, sprinting ability, and COD speed through PT training, whereas no improvements in sprinting ability and COD speed were found for players aged 15 to 17.99. Male and female youth basketball players could improve their jumping through PT, in contrast, straight-line sprinting ability and COD speed were significantly improved only by male youth basketball players, and balance ability was significantly improved only by female youth basketball players. 3) Regarding different training protocols, high-frequency PT (>2 times/week) with a low-volume (jumping ≤150 times/week) and Single-type PT (one specific movement) improved only jumping ability. In contrast, low-frequency PT (1–2 times/week) with a high-volume (jumping >150 times/week) and mixed-type PT (varied jumping drills) protocols significantly improved jumping, linear sprinting, COD speed, and balancing abilities.

**Conclusion:**

PT can enhance the jumping, linear sprinting, COD speed, and balance of youth basketball players, but it does not affect lower limb strength. It is recommended that coaches make full use of the training-sensitive periods of young athletes by incorporating low-frequency, high-volume, and mixed-type PT into their regular training routines over the long term.

## 1 Introduction

Basketball is an intermittent team sport marked by intense physical contact and rapid transitions between offense and defense. During a game, players repeatedly engage in high-intensity activities such as sprinting, shuffling, jumping, accelerations, decelerations, and changes of direction (CODs) (Pernigoni et al., 2021). As the competitive level of basketball continues to escalate globally, the performance of athletes has become one of the critical determinants of success in the sport. In recent years, the emergence of advanced training methods and techniques has underscored the importance of scientific training approaches in basketball. Plyometric training (PT), which consists of exercises exploiting the stretch-shortening cycle (SSC) (Sole, 2017; Markovic and Mikulic, 2010), has gained increasing attention and application among coaches due to its beneficial effects on athletic performance (Weldon et al., 2022). The fundamental principle of this training modality is based on the exploitation of the SSC of skeletal muscles, which encompasses three distinct phases (Ramírez-delaCruz et al., 2022b): The initial phase involves the eccentric contraction, where the tendons resist an external load, undergo passive elongation, induce a protective inhibition, and store elastic potential energy. This is followed by an isometric contraction phase, during which the stored elastic potential energy is transferred to the skeletal muscles. Finally, in the concentric contraction phase, the inclusion of elastic potential energy enhances the power output during the shortening action of the skeletal muscles. At the same time, PT can enhance motor unit recruitment capacity and synchronization, improving muscle adaptability and response to force, which in turn facilitates the development of muscular strength, power, and overall body coordination (Markovic and Mikulic, 2010; Cormie et al., 2011). Therefore, the nature of PT aligns well with the physiological demands and movement characteristics inherent in the sport of basketball (Asadi et al., 2017; Arede et al., 2019).

However, the nervous and muscular systems are not fully developed during youth. Differences in neural conduction velocity, control capabilities, and muscle architecture between youths and adults significantly impact the control and coordination of their SSC (Radnor et al., 2018; Lloyd and Oliver, 2012) summarize the training sensitive periods for athletes aged 2–21 years based on growth and development characteristics in their YPD model. They identify the critical periods for athletic development as ages 12 to 16 for males and 11 to 15 for females, during which agility, speed, strength, and endurance exhibit heightened sensitivity to training. Previous research indicates that PT can enhance vertical jump performance and sprint speed in prepubescent boys (Kotzamanidis, 2006), as well as jumping, COD speed, and balance abilities in adolescent female basketball players (Bouteraa et al., 2020), with meta-analytic results also indicating that PT can improve strength and jumping capabilities in youth athletes (Behm et al., 2017; Chen et al., 2023; Moran et al., 2017). However, the specific training effects of PT on youth athletes at different stages of development remain unclear, particularly in the absence of a comprehensive understanding of the maturation cycle of the SSC in youths. It is uncertain whether the training effects of PT have been maximized under these conditions. Furthermore, existing research has demonstrated that PT can enhance the athletic abilities of adolescent basketball players by improving the utilization level of the SSC (Asadi et al., 2017; Arede et al., 2019). However, further discussion is required to discern the differential impacts of various PT protocols on the relevant abilities of adolescents. Moreover, a meta-analysis focusing on the effects of PT on the athletic performance of youth basketball players has yet to be reported. Notably, the existence of an optimal training regimen involving combinations of different frequencies, volumes, types, and durations of PT remains to be determined. Therefore, investigating the influence of PT on athletic performance across various growth stages in adolescents and devising safe and effective training protocols tailored to specific performance outcomes constitute pressing issues that need to be addressed. The training effects of PT across different ages and sexes, the differential impacts of various frequencies, volumes, types, and durations of PT on the athletic performance of youth basketball players, and whether the influence of PT on athletic performance is selective all warrant further exploration.

In summary, the present study conducts a meta-analysis of experimental research concerning the effects of PT on the athletic performance of professional and amateur youth basketball players. The objective is to explore the impact of PT on athletic performance and provide a theoretical foundation for the practical application of PT in the training of youth basketball players.

## 2 Methods

This study was conducted in accordance with the Preferred Reporting Items for Systematic Reviews and Meta-Analyses (PRISMA) guidelines (Page et al., 2021), and the PRISMA checklist is provided in (Supplementary Appendix). This meta-analysis was registered on the PROSPERO platform on 28 October 2023, with the registration number CRD42023473515.

### 2.1 Search strategy and selection criteria

Based on previous meta-analyses examining PT in other sports (Chen et al., 2023; Ramirez-Campillo et al., 2020), the search strategy was meticulously detailed, tested, and refined. The detailed search strategies for each individual database are provided in Table 1. Following the identification of relevant search terms, two researchers (ZJY and WX) conducted comprehensive searches across “Web of Science” “Embase” “Cochrane Library” and “PubMed”. The search scope covered from the inception of each database up to 2 October 2023, and subscriptions ensure that newly published literature is promptly supplemented. Following the formal systematic searches, additional hand searches were conducted using the authors’ personal libraries and known published reviews, systematic reviews, and meta-analyses. Duplicate articles were removed after the initial search phase. Subsequent screening of search results was conducted based on predefined inclusion and exclusion criteria. Initially, two researchers (LH and WX) independently evaluated titles and abstracts, followed by thoroughly examining full-text articles. Any disparities in screening results were resolved through discussion with a third reviewer (WW), ensuring consensus was reached through iterative pre-screening and deliberation.

**TABLE 1:** Detailed study retrieval strategies.

	(P) Basketball players (I) Plyometric training	Results
PubMed (1986 – October 2023	(“plyometric training” [Title/Abstract] OR “plyometric exercise*” [Title/Abstract] OR “plyometric drill*” [Title/Abstract] OR “plyometr*” [Title/Abstract] OR “ballistic training” [Title/Abstract] OR “explosive” [Title/Abstract] OR “force-velocity” [Title/Abstract] OR “stretch-shortening cycle” [Title/Abstract] OR “stretch-shortening exercise” [Title/Abstract] OR “complex training” [Title/Abstract] OR “jump training” [Title/Abstract]) AND (“basketball” [Title/Abstract] OR “basketball player*” [Title/Abstract] OR “basketball athlete*” [Title/Abstract])	177
Web of Science (1993 – October 2023	AB = (“plyometric training” OR “plyometric exercise*” OR “plyometric drill*” OR “plyometr*” OR “ballistic six” OR “ballistic training” OR “explosive” OR “force-velocity” OR “stretch-shortening cycle” OR “stretch-shortening exercise” OR “complex training” OR “jump training”) AND AB = (“basketball” OR “basketball player*” OR “basketball athlete*”)	255
EBSCOhost (1962 – October 2023)	AB = (“plyometric training” OR “plyometric exercise*” OR “plyometric drill*” OR “plyometr*” OR “ballistic six” OR “ballistic training” OR “explosive” OR “force-velocity” OR “stretch-shortening cycle” OR “stretch-shortening exercise” OR “complex training” OR “jump training”) AND AB = (“basketball” OR “basketball player*” OR “basketball athlete*”)	585
Cochrane Library (1986 – October 2023)	(“plyometric training” OR “plyometric exercise*” OR “plyometric drill*” OR “plyometr*” OR “ballistic six” OR “ballistic training” OR “explosive” OR “force-velocity” OR “stretch-shortening cycle” OR “stretch-shortening exercise” OR “complex training” OR “jump training”) AND (“basketball” OR “basketball player*”OR “basketball athlete*”)	104
Total		1,121

### 2.2 Inclusion and exclusion criteria

The criteria for inclusion are as follows: 1) Participants must be either professional or amateur basketball players and aged <18 years old; 2) The intervention method should involve PT and must include at least one bilateral or unilateral training exercise capable of stimulating the SSC; 3) The control group participating in regular basketball training; 4) Outcome measures must encompass at least one sports performance metric, such as strength, jump height, speed, or balance; 5) Studies must be controlled trials; 6) Given the potential challenges of translating articles from different languages and the fact that 99.6% of the PT literature is published in English (Ramirez-Campillo et al., 2022), this meta-analysis includes only articles written in English. Additionally, this study excluded research that met the following criteria: 1) Participants with movement disorders or other illnesses, athletes from other sports, or aged ≥18 years old; 2) Acute exercise interventions; 3) Incomplete data that prevented the direct or indirect acquisition of pre and post-test data; 4) non-interventional clinical trials, such as protocols, review studies, cohort studies, case-control studies, conference papers, and book chapters.

### 2.3 Data extraction

Data extraction was independently performed by two authors (ZJY and WX). Any discrepancies were resolved through discussion between the authors. If consensus could not be reached, a third author made the final decision (WW). The extracted data included study characteristics (first author’s name, publication year) and participant demographics (sample size, age, sex, competitive level). Specifically, competitive level was determined according to previous research (Russell et al., 2021) as follows: Level 1) untrained or sedentary participants; Level 2) habitually active, physically fit, or recreationally-trained participants; Level 3) trained and competitive players; Level 4) highly-trained and competitive players; or Level 5) professional players. intervention details (overall length, frequency, total jumps, type), investigated measures, and pre-and post-test results (relevant statistical data for estimating effect sizes). To address missing data, we contacted the authors via email on at least three occasions. When no response was received, information from studies that presented only graphical data was extracted using WebPlotDigitizer (v4.3, Ankit Rohatgi; https://apps.automeris.io/wpd/), which has shown acceptable validity and reliability in extracting graph data (Drevon et al., 2017).

### 2.4 Data coding and management

In this study, to explore the optimal age stage for adolescents to participate in PT, participants were divided into three age groups: 5–10.99 years, 11–14.99 years, and 15–17.99 years (Lloyd and Oliver, 2012). In terms of the training program, to investigate the effectiveness of different PT protocols, this study categorizes PT into two types: single-type PT and mixed-type PT. Single-type PT refers to training programs that consist of only one specific exercise, such as performing only vertical jumps. In contrast, mixed-type PT includes a combination of two or more jump exercises, such as performing vertical jumps, horizontal jumps, bilateral jumps, unilateral jumps, repeated jumps, and non-repeated jumps simultaneously (Ramirez-Campillo et al., 2022). Regarding athletic performance, we decided to categorize performance metrics into two separate tiers, with the aim of reflecting the different physiological and biomechanical indices involved in basketball-related athletic performance (Ben Abdelkrim et al., 2007; Delextrat and Cohen, 2008; Shin, 2008). Primary performance indicators were classified into lower body strength, jumping ability, straight sprinting ability, COD speed, and balance. Within secondary performance indicators, jumping ability was further subdivided into horizontal (e.g., long jump) and vertical (e.g., rebound jump, standing knee-up jump). Straight sprinting ability was categorized based on test distances into two ranges: 5–10 m and 10–30 m. COD is divided into velocity-oriented COD (COD Angle ≤90°, such as V-cut test) and force-oriented COD (COD Angle >90°, such as Illinois Agility test) (Nygaard Falch et al., 2019). Balance was classified into static (e.g., single-leg stand) and dynamic balance (e.g., Y-balance test). All data were recorded and stored using Microsoft Excel (Microsoft Corporation, Redmond, WA, United States), specific classification results are detailed in Appendix 2, Tables 4-8.

### 2.5 Study quality assessment and quality of evidence

Two authors (ZJY and WX) utilized the Physiotherapy Evidence Database scale (PEDro) (Maher et al., 2003) to assess the quality of studies. Any discrepancies between the authors’ evaluations were resolved through discussion to achieve consensus. The PEDro scale comprises 11 criteria: eligibility, randomization, allocation concealment, baseline comparability, blinding of participants, therapists, and assessors, adequacy of follow-up, intention-to-treat analysis, and between-group comparisons, including point estimates and variability. Each criterion (2–11) is assigned 1 point, with a maximum score of 10. The quality of studies was categorized based on the total score: <4 points denoted poor quality, 4 to 5 points indicated fair quality, 6 to 8 points signified good quality, and 9 to 10 points represented excellent quality.

The certainty of the evidence was evaluated by two authors (ZJY and WX) using the Grading of Recommendations, Assessment, Development, and Evaluation (GRADE) methodology, which categorizes evidence as very low, low, moderate, or high (Guyatt et al., 2011; Zhang et al., 2019a; Zhang et al., 2019b). The evidence was initially rated as high for each outcome, and was later downgraded based on the following criteria: 1) risk of bias in studies: if the median PEDro scores were moderate (<6), the evidence was downgraded by one level; 2) indirectness: low risk of indirectness was assumed by default due to the specificity of populations, interventions, comparators, and outcomes ensured by the inclusion/exclusion criteria; 3) risk of publication bias: the evidence was downgraded by one level if there was suspected publication bias (Egger’s test *p* < 0.05); 4) inconsistency: the evidence was downgraded by one level when statistical heterogeneity (I^2^) was high (>50%); 5) Imprecision: the evidence was downgraded by one level if the number of participants available for comparison was small (<800) (Deng et al., 2023; Guyatt et al., 2021).

### 2.6 Statistical analysis

First, we addressed the issue of missing data in the included studies. If the standard deviations (SDs) were not directly provided in the literature, we used RevMan 5.4.1 software to calculate the SD based on the standard errors (SEs), confidence intervals (CIs), or statistical values (such as t-values or *p*-values).

Subsequently, we performed some data adjustment, 1) In cases where studies employed reverse scaling (where a lower value indicated a better outcome, such as a 20 m run time), we adjusted the mean for each group by multiplying it by −1. 2) We entered multiple intervention arms of the same study as separate interventions in the meta-analysis. We divided the sample size of the control group by the number of intervention arms in the study to avoid overestimating the pooled effect size. We left the means and standard deviations unchanged, as recommended in the Cochrane Handbook for Systematic Reviews of Interventions (Higgins and Green, 2011). 3) In meta-analyses, changes in the mean and the SD of these changes are often considered missing data points (Higgins et al., 2021). Previous systematic reviews have highlighted the challenges posed by this missing data for conducting comprehensive meta-analyses (Shida et al., 2021; Yagiz et al., 2021). To address this issue, specific formulas have been established (Borenstein et al., 2009; Higgins and Green, 2011) (see Equations 1, 2).

$$\left(\right){\text{Mean}}_{change}={\text{Mean}}_{final}-{\text{Mean}}_{baseline}$$ (1)

$$\left(\right)SD_{change}=\sqrt{S{D²}_{baseline}+S{D²}_{final}-2R\times SD_{baseline}\times SD_{final}}$$ (2)

In these formulas, ${\text{Mean}}_{change}$ and $SD_{change}$ represent the change in the mean and the standard deviation of the change in the mean, respectively, while ${\text{Mean}}_{baseline}$ and $SD_{baseline}$ represent the pre-test mean and its standard deviation, ${\text{Mean}}_{final}$ and $SD_{final}$ represent the post-test mean and its standard deviation. The correlation R between the baseline and final measurements is often not reported in studies. Previous meta-analyses related to PT and jump performance have shown that this correlation ranges from 0.81 to 0.84 (Markovic, 2007). However, since this study includes multiple performance outcome measures, a conservative estimate of R is set at 0.7, consistent with practices in previous systematic reviews (Yagiz et al., 2022).

Finally, statistical analysis was conducted. The Kappa scores calculated using IBM SPSS Statistics 25.0 (SPSS, Chicago, Illinois, United States) assessed the consistency between the reviewers for abstract and full-text screening. The Kappa scores were interpreted as excellent (≥0.75), good (0.60–0.74), fair (0.40–0.59), or poor (<0.40) (Rigby, 2000). Meta-analysis was performed using STATA 16.0 software, with the active control group participating in training being considered the comparison group for this meta-analysis. Due to the different outcome measurement methods in the included studies, the study used standardized mean differences (SMD) and 95% CI to estimate the summary effect size (ES). ES were interpreted as small (0.2–0.5), medium (0.5–0.8), or large (>0.8) (Cohen, 1988). Heterogeneity was assessed using the Q statistic and I^2^. I^2^ results were interpreted as low (<25%), moderate (50%) and high (>75%) (Higgins et al., 2003). When statistical heterogeneity was not detected (*p* > 0.05 in the Q statistic and I^2^ <50%), a fixed-effect model was employed for the meta-analysis; otherwise, a random-effects model was selected (DerSimonian and Laird, 1986). Publication bias was assessed using the extended Egger’s test and funnel plots. A significance level of *p* < 0.05 in Egger’s test indicated significant publication bias (Egger et al., 1997). Additionally, sensitivity analysis was performed by sequentially omitting each study to evaluate whether the summary estimates were unduly influenced by any single study (Tobias, 1999). If the sensitivity analysis results were consistent with the meta-analysis results, it would enhance the credibility of the meta-analysis; otherwise, results should be interpreted with caution. Figures and charts were generated using R-evolution 4.2.1.

## 3 Results

### 3.1 Characteristics and assessment of included studies

**FIGURE 1:**
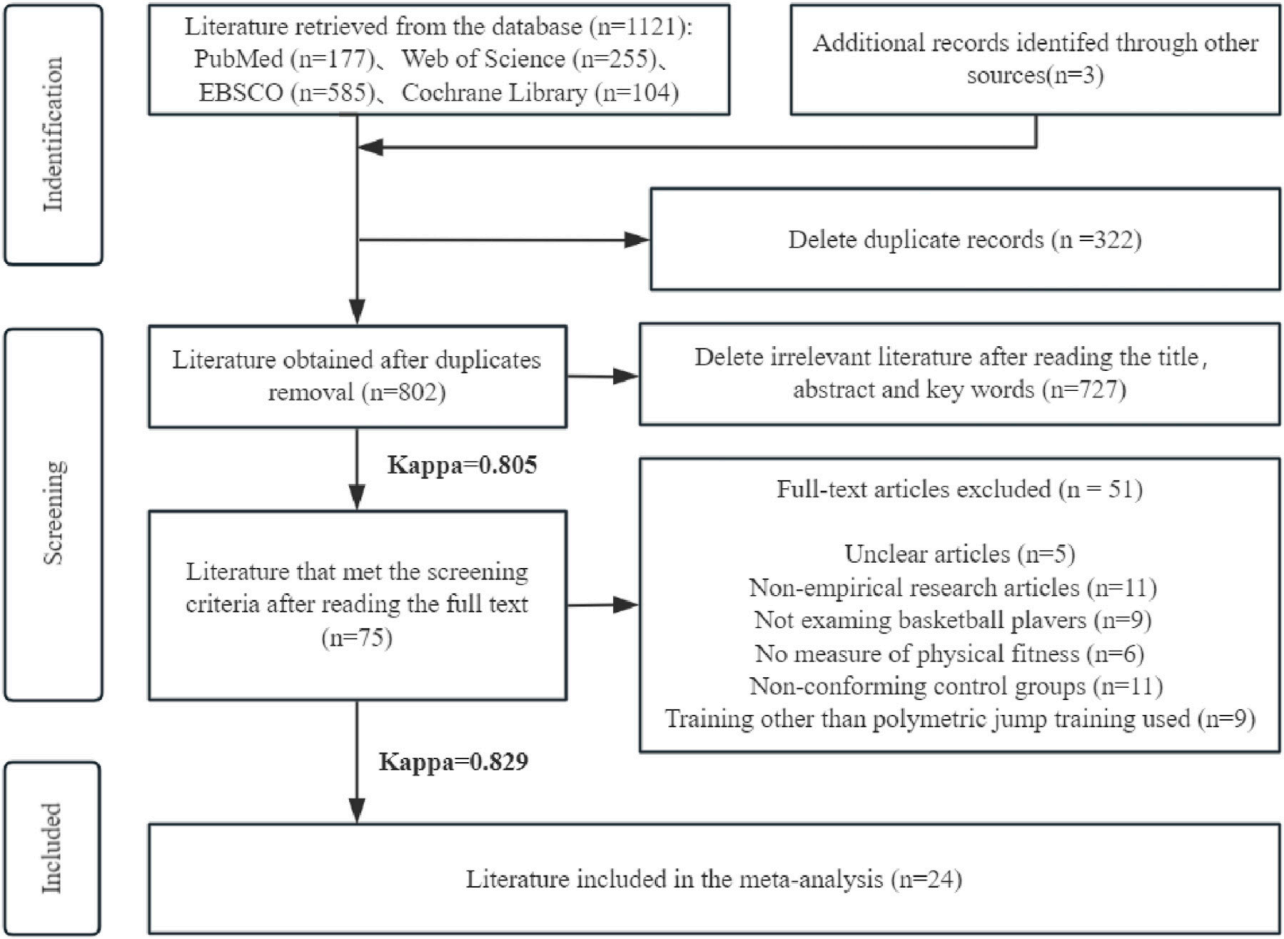
Literature search and screening process.

Our search across all four databases yielded 1,121 articles, of which 24 were ultimately included in the study. The inter-rater agreement, measured by Cohen’s Kappa coefficient >0.75, indicates a high level of consistency in the literature screening results (Supplementary Table S1, S2; Table 1 and Figure 1).

Within the included studies, four articles explored lower limb strength, twenty-one investigated jumping ability, eleven examined linear sprint capacity, twelve addressed COD speed, and four focused on balance ability. The cumulative number of experimental subjects was 738, with 399 in the experimental group and 344 in the control group. Male participants constituted 74.96% of the sample. The average height was 1.65 ± 0.14 m, and the average weight was 55.35 ± 13.68 kg. The average training duration was 7.4 ± 1.91 weeks, with a frequency of 2.57 ± 0.57 sessions per week, totaling 1,317 ± 840 jumps. Specific characteristics of the literature are detailed in Table 2.

**TABLE 2:** Summary of characteristics of all studies meeting the inclusion criteria. (n = 24).

Study	Population characteristics	Interventions	Control	Characteristics of intervention	Investigated measures	Outcome		PEDro
				Dur/Freq/Type/TJ		Time (Pre vs Post)	Groups (E.G., vs CG)	
Sidki Adigüzel and Günay (2016)	Sex = M; CL = Level 4 E.G.,: N = 15.A = 15; H=NR, BM = NR CG: N = 15.A = 18; H=NR, BM = NR	PT + BT	BT	Dur = 8 Freq = 3 Type = Mixed TJ = 1,449	①:60°,180° Right (Hamstring) peak power; 60°,180° Left (Hamstring) peak power; 60°,180° Right (Quadricep) peak power; 60°,180° Left (Quadricep) peak power ②: CMJA; CMJ; SJ	E.G.,: 60°Right (Hamstring)↑; 60°Left (Quadricep)↑; 180°Right (Hamstring)↑; 180°Left (Hamstring)↑; 180°Right (Quadricep)↑; 180°Left (Quadricep)↑; CMJA↑; CMJ↑; SJ↑; other↔ CG: 60°Right (Hamstring)↑; 60°Right (Quadricep)↑; 60°Left (Quadricep)↑; CMJA↑; CMJ↑; SJ↑; other↔	60°Right (Hamstring) peak power↑; 60°Right (Quadricep) peak power↑; 180° Left (Hamstring) peak power↑; 180°Right (Quadricep) peak power↑ CMJA↑; CMJ↑; SJ↑	4
Amato et al. (2018)	Sex = M; CL = Level 3 E.G.,: N = 12; A = 12years; H = 1.53; BM = 50.3 CG: N = 11; A = 11year; H = 1.57; BM = 47.1	PT + BT	BT	Dur = 6 Freq = NR Type = Mixed TJ = 440	②: CMJ; SJ; DJ ③: V_25m_ ④:*t*-test	E.G.,: all↑ CG: CMJ↓; DJ↓; *t*-test↑; other↔	NR	4
Andreji (2012)	Sex = M; CL = Level 3 E.G.,: N = 10; A = 12.6years; H = 1.71; BM = 58.0 CG: N = 11; A = 12.5years; H = 1.73; BM = 62.0	PT + RT	RT + BT	Dur = 6 Freq = 2 Type = Mixed TJ = 164	②: CMJA; SLJ ③:V_20m_; V_4×15m_	E.G.,: all↑ CG: all↔	all ↑	6
Arede et al. (2019)	Sex = M; CL = Level 4 E.G.,: N = 9; A = 14.2; H = 1.65; BM = 56.2 CG: N = 7; A = 14.9; H = 1.75; BM = 62.6	PT + BT	BT	Dur = 8 Freq = 4 Type = Mixed TJ = NR	②: CMJ; SJ ③: V_10m_ ④: Pro agility test	E.G.,: SJ↑; V_10m_↑; other↔ CG: CMJ↑; other↔	V_10m_↑; other↔	3
Attene et al. (2015)	Sex = F; CL = Level 4 E.G.,: N = 18; A = 14.8; H = 1.63; BM = 51.9 CG: N = 18; A = 15.2; H = 1.65; BM = 57.5	PT + BT	BT	Dur = 6 Freq = 2 Type = Mixed TJ = 1,120	②: CMJ; SJ	E.G.,: all↑ CG: all↑	all ↔	7
Aztarain-Cardiel et al. (2024)	Sex = M; CL = Level 4 E.G.,1: N = 10 A = 14.5; H = 1.81, BM = 69.0 E.G.,2: N = 11 A = 15.1; H = 1.81, BM = 70.2 CG: N = 10; A = 15.3; H = 1.87, BM = 74.8	PT + BT PT + BT	BT	Dur = 6 Freq = 4 Type = Single (Abalakov Jump) TJ = E.G.,1:2368 ; E.G.,2:1,184	②: CMJ; SJ; HJ ③: V_20m_ ④: V cut test	E.G.,1: CMJ↑; SJ↑ other↔ E.G.,2: CMJ↑; SJ↑ HJ↑; other↔ CG: all↔	CMJ↑; SJ↑ HJ↑; other↔	5
Bouteraa et al. (2020)	Sex = F; CL = Level 4 E.G.,: N = 16; A = 16.4; H = 1.68; BM = 56.6 CG: N = 10; A = 16.5; H = 1.68; BM = 55.6	PT + BT	BT	Dur = 8 Freq = 2 Type = Mixed TJ = 1,588	②: CMJ; SJ; DJ ③: V_5m_; V_10m_; V_20m_ ④: Modified Illinois test ⑤: Y-balance test; Stork balance test	E.G.,: DJ↑; Modified Illinois test↑; Y-balance test↑; Stork balance test↑; other↔ CG: all↔	CMJ↑; DJ↑; V_5m_↑; V_10m_↑; Illinois test↑; other↔	5
Brown et al. (1986)	Sex = M; CL = Level 4; N = 26; A = 15; 1.81 ± 0.08; BM = 67.9 ± 8.1	PT + BT	BT	Dur = 12 Freq = 2–3 Type = Single (DJ) TJ = 1,080	②: CMJA; CMJ	E.G.,: all↑ CG: CMJA↑; CMJ↔	all↑	5
Chouhan et al. (2022)	Sex = M; CL = Level 3 E.G.,: N = 45; A = 16.5; H=NR; BM = NR CG: N = 45; A = 16.9; H=NR; BM = NR	PT + BT	Pilates + BT	Dur = 6 Freq = 3 Type = Mixed TJ = 635	②: Sargent jump test ③: V_20m_	E.G.,: Sargent jump test↑; V_20m_↔ CG: Sargent jump test↑; V_20m_↔	Sargent jump test↑; V_20m_↔	5
Cigerci and Genc (2020)	Sex = M; CL = Level 2 E.G.,: N = 10; A = 15.9; H = 1.78; BM = 69.5 CG: N = 10; A = 15.4; H = 1.74; BM = 64.9	PT + BT	BT	Dur = 8 Freq = 3 Type = Mixed TJ = 3024	②: SLJ; VJ ③: V_10m_; V_20m_ ④: Lane agility test ⑤: Star excursion balance test (right/lift points)	E.G.,: SLJ↑; VJ↑; V_10m_↑; V_20m_↑; Lane agility test↑; other↔ CG: SLJ↑; VJ↑; other↔	SLJ↑; VJ↑; V_10m_↑; V_20m_↑; Lane agility test↑; other↔	4
Rafael et al. (2017)	Sex = M; CL = Level 3 E.G.,: N = 18; A = 15.2; H = 1.80; BM = 72.6 CG: N = 21; A = 16.4; H = 1.76; BM = 72.8	PT + BT	RT + BT	Dur = 8 Freq = 2 Type = Mixed TJ = 1,440	④: *t*-test	E.G.,: *t*-test↑ CG: *t*-test↑	*t*-test↑	6
Fontenay et al. (2013)	Sex = F; CL = Level 3 E.G.,: N = 9; A = 15.5; H=NR; BM = NR CG: N = 8; A = 15.5; H=NR; BM = NR	PT + BT	BT	Dur = 8 Freq = 2 Type = Single (Hop) TJ = NR	②: DJ	E.G.,: DJ↑ CG: DJ↔	DJ↑	3
Gottlieb et al. (2014)	Sex = M; CL = Level 4; A = 16.3; H = 185.3; BM = 78.2 E.G.,: N = 9 CG: N = 10	PT + BT	SST + BT	Dur = 6 Freq = 2 Type = Mixed TJ = 1,080	②: CMJ; 6-bound jump distance ③: V_20m_ ④: 2 × 5 m shuttle run	E.G.,: all ↔ CG:6-bound jump distance↑; V_20m_↑; other ↔	all↔	5
Haghighi et al. (2023)	Sex = F; CL = Level 5 E.G.,: N = 8; A = 14.6; H = 168.3; BM = 61.7 CG: N = 8; A = 15.1; H = 165.8; BM = 56.7	PT + BT	BT	Dur = 6 Freq = 2 Type = Mixed TJ = 1,636	③: V_20m_ ④: *t*-test	E.G.,: all↑ CG: all↔	V_20m_↑; other ↔	7
Hernández et al. (2018)	Sex = M; CL = Level 2 E.G.,1: N = 6; A = 10; H = 1.41; BM = 36.3 E.G.,2: N = 7; A = 11; H = 1.41; BM = 38.3 CG: N = 6; A = 9.7; H = 1.44; BM = 39.4	PT + BT PT + BT	BT	Dur = 7 Freq = 2 Type = Mixed TJ = 1,044	②: CMJ; DJ ③: V_30m_ ④: *t*-test	E.G.,1: all↑ E.G.,2: all↑ CG: all↔	E.G.,1: all↑ E.G.,2: all↔	8
Latorre Román et al. (2017)	Sex = M/F; CL = Level 2 E.G.,: N = 30; A = 8.72; H = 1.33; BM = 30.6 CG: N = 28; A = 8.72; H = 1.40; BM = 35.1	PT + BT	BT	Dur = 10 Freq = 2 Type = Single (DJ) TJ = 1920	②: SJ; CMJ; SLJ; DJ ③: V25 m ④: *t*-test	E.G.,: SLJ↑; CMJ↑; DJ↑; V_25m_↑; *t*-test↑; other↔ CG: SLJ↑; CMJ↑; DJ↑; V_25m_↑; other↔	V_25m_↑; *t*-test↑; other↔	5
Matavulj et al. (2022)	Sex = M; CL = Level 4; A = 15–16; H=NR; BM = NR E.G.,1: N = 11 E.G.,2: N = 11 CG: N = 11	PT + BT PT + BT	BT	Dur = 6 Freq = 3 Type = Single (DJ) TJ = 540	①: Knee extensor strength ②: CMJ	E.G.,1: CMJ↑; other↔ E.G.,2: CMJ↑; other↔ CG: all↔	CMJ↑; other↔	4
McLeod et al. (2009)	Sex = F; CL = Level 2 E.G.,: N = 27; A = 15.6; H = 1.71; BM = 58.9 CG: N = 23; A = 16; H = 1.72; BM = 62.3	PT + BT	BT	Dur = 6 Freq = 2 Type = Mixed TJ = 288	⑤: Star excursion balance test; Balance Error Scoring System composite score	E.G.,: Balance Error Scoring System composite score↑; other↔ CG: all↔	Star excursion balance test↑; other↔	4
Meszler and Váczi (2019)	Sex = F; CL = Level 4 E.G.,: N = 9; A = 15.8; H = 1.76; BM = 63.5 CG: N = 9; A = 15.7; H = 1.77; BM = 66.1	PT + BT	BT	Dur = 7 Freq = 2 Type = Mixed TJ = 1,027	①: Knee extensor strength ②: CMJ ④: *t*-test; Illinois test ⑤: Balance with eyes open, unilateral	E.G.,: CMJ↓; others↔ CG: knee extensor strength↑; others↔	CMJ↓; others↔	4
Pamuk et al. (2022)	Sex = M; CL = Level 5 E.G.,1: N = 11; A = 15.5; H = 1.79; BM = 68.6 E.G.,2: N = 12; A = 15.7; H = 1.84; BM = 73.9 CG: N = 12; A = 15.5; H = 1.84; BM = 70.2	E.G.,1:PT + BT E.G.,2:PT + BT	BT	Dur = 12 Freq = 3 Type = Mixed TJ = NR	①Isokinetic peak moment results of the knee during flexion and extension (60°/s,180°/s,300°/s-left); Isokinetic peak moment results of the knee during flexion and extension (60°/s,180°/s,300°/s-right) ②: Jumping height	E.G.,: 180°/s-right↑; 180°/s-lift↑; 300°/s-right↑; 300°/s-lift↑; other↔ CG: 300°/s-lift↑; other↔	all↔	5
Santos and Janeira (2008)	Sex = M; CL = Level 3 E.G.,1: N = 14; A = 15; H = 1.73; BM = 62.2 CG: N = 10; A = 14.5; H = 1.73; BM = 61.1	PT + BT	BT	Dur = 10 Freq = 2 Type = Mixed TJ = 1,656	②: CMJA; CMJ; SJ; DJ	E.G.,: all↑ CG: CMJA↑; CMJ↑; SJ↑; other↔	all↑	5
Santos and Janeira (2009)	Sex = M; CL = Level 2 E.G.,: N = 8; A = 14; H = 1.77; BM = 75.6 CG: N = 7; A = 15; H = 1.74; BM = 69.3	PT + BT	BT	Dur = 10 Freq = 2 Type = Mixed TJ = 768	②: CMJA; CMJ; SJ; DJ	E.G.,: all↔ CG: all↔	all↔	4
Santos and Janeira (2011)	Sex = M; CL = Level 3 E.G.,: N = 15; A = 14.7; H = 1.75; BM = 72.7 CG: N = 10; A = 14.2; H = 1.73; BM = 61.1	PT + BT	BT	Dur = 10 Freq = 2 Type = Mixed TJ = 1,694	②: CMJ; SJ; DJ	E.G.,: CMJ↑; SJ↑; DJ↔ CG: CMJ; other↔	SJ↑ DJ↑ other↔	4
Zribi et al. (2014)	Sex = M, CL = Level 2 E.G.,: N = 25; A = 12.1; H = 1.56; BM = 41.1 CG: N = 26; A = 12.2; H = 1.55; BM = 41.2	PT + BT	BT	Dur = 9 Freq = 2 Type = Mixed TJ = 1,440	②: CMJA; CMJ; SJ; 5-bound jump distance ③: V_5m_; V_30m_	E.G.,: CMJA↑; CMJ↑; SJ↑; V_5m_↑; V_30m_↑; other↔ CG: all↔	CMJA↑; CMJ↑; SJ↑; V_5m_↑; V_30m_↑; other↔	5

Note: Competitive level was classified according to Russell et al. (2021): Level 1) untrained or sedentary participants; Level 2) habitually active, physically fit or recreationally-trained participants; Level 3) trained and competitive players; Level 4) highly-trained and competitive players; Level 5) professional players.

Symbols: ①, Lower limb strength; ②, Jump; ③, Linear sprinting; ④, Agility; ⑤, Balance; ↑, significantly positive effect (*p* ≤ 0.05); ↓, significantly negative effect (*p* ≤ 0.05); ↔, no effect (*p* > 0.05).

Abbreviations NR, not reported; M, male; F, female; CL, competitive level; A, age (years); H, height (m); BM, body mass (kg); PT, plyometric training; BT, basketball training; E.G., experimental group; CG, control group; Dur, duration of training (weeks); Freq, frequency of training (session/week); TJ, total jumps; CMJA, countermovement jump with arm; CMJ, countermovement jump; SJ, squat jump; DJ, drop jump; SLJ, stand long jump; HJ, high jump; VJ, vertical jump; V_5m_, speed in 5 m; V_10m_, speed in 10 m; V_15m_, speed in 15 m; V_20m_. speed in 20 m; V_25m_, speed in 25 m; V_30m_, speed in 30 m.

The quality assessment of the included literature yielded an average score of 4.87 ± 1.22 points, indicating a moderate overall quality of the studies. The primary risk of bias arose from the lack of blinding of participants, personnel, and outcome assessors during the grouping process and training interventions (Supplementary Appendix S2; Table 2).

### 3.2 Results of Meta-analysis

#### 3.2.1 Effect of PT on lower limb strength

Four studies (n = 93 participants) assessed the lower limb strength of youth basketball players, revealing moderate heterogeneity among the studies (I^2^ = 34.6%, *p* = 0.053). Therefore, a fixed-effects model was employed for the analysis. The results indicated that PT did not significantly enhance the lower limb strength of youth basketball players (SMD = 0.07, 95% CI: −0.12 to 0.25, *p* = 0.479) (Supplementary Appendix S2; Supplementary Figure S1).

#### 3.2.2 Effect of PT on jumping

**FIGURE 2:**
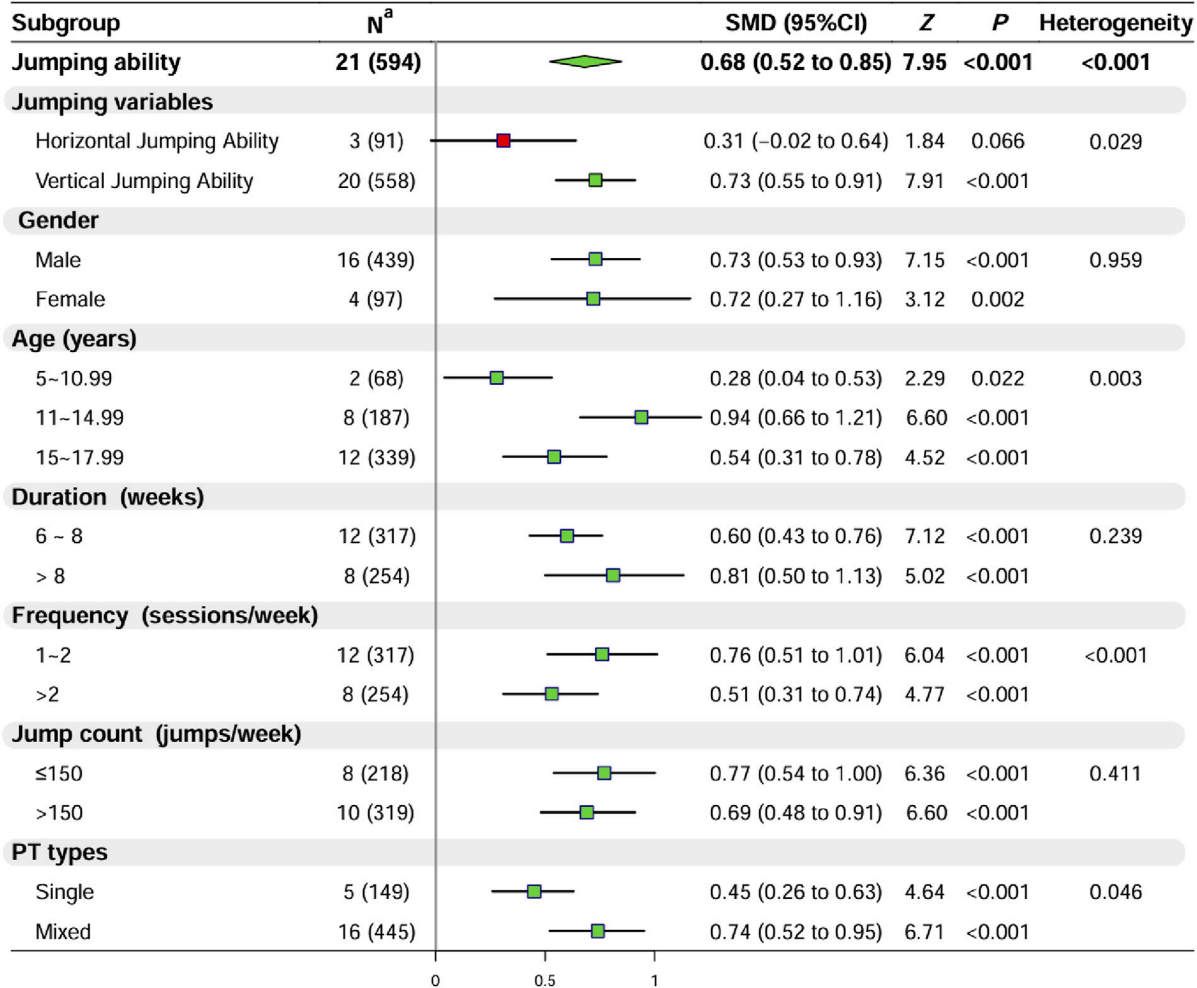
Results of the Meta-Analysis and Subgroup Analysis on Jumping Ability. Note: 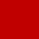 mean nonsignificant (*p* > 0.05) and 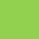 mean significant (*p* < 0.05) improvement were observed in the experimental group after plyometric jump training compared with the control group; ^a^ Data denote the number of included studies (total number of participants included). Abbreviations: SMD, standardized mean difference; CI, confidence interval; PT, plyometric training.

Twenty-one studies (n = 594) assessed the jumping ability of youth basketball players, revealing moderate heterogeneity among the studies (I^2^ = 59.9%, *p* < 0.001). Therefore, a random-effects model was utilized for the analysis. The results indicated a statistically significant difference compared to the control group (SMD = 0.68, 95% CI: 0.52 to 0.85, *p* < 0.001) (Supplementary Appendix S2; Supplementary Figure S2; Figure 2), suggesting that PT significantly improves the jumping ability of youth basketball players. Further analysis of horizontal and vertical jumping capabilities revealed that PT significantly enhances vertical jumping ability in youth basketball players (*p* < 0.001), while the increase in horizontal jumping ability was insignificant (*p* = 0.066).

Regarding participant characteristics: 1) PT enhanced the jumping ability of male and female basketball players (*p* < 0.01); 2) PT significantly improved the jumping ability of basketball players aged 5 to 10.99 (*p* < 0.05), 11 to 14.99 (*p* < 0.001) and 15–17.99 years (*p* < 0.001). Concerning training protocols: 1) Both 6–8 weeks (*p* < 0.001) and >8 weeks (*p* < 0.001) of PT significantly improved the jumping ability of youth basketball players; 2) Both training frequencies of ≤2 sessions per week (*p* < 0.001) and >2 sessions per week (*p* < 0.001) significantly improved jumping ability; 3) Both PT with jumping repetitions ≤150 times per week (*p* < 0.001) and >150 times per week (*p* < 0.001) significantly enhanced jumping ability; 4) Both mixed-type (*p* < 0.001) and single-type (*p* < 0.001) jump training significantly improved the jumping ability of youth basketball players.

#### 3.2.3 Effect of PT on linear sprint

**FIGURE 3:**
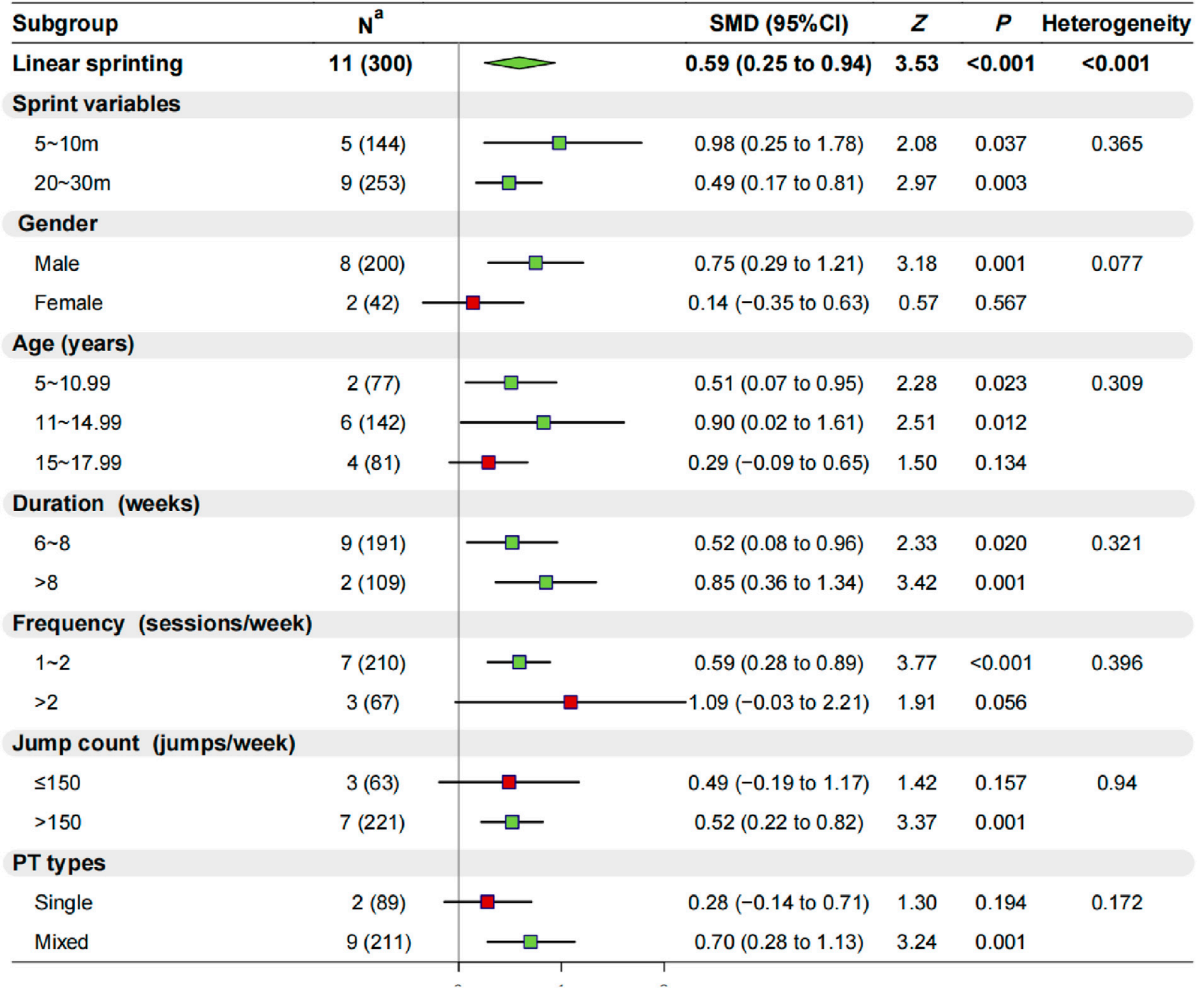
Results of Meta-Analysis and Subgroup Analysis on linear sprinting. Note: 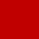 mean nonsignificant (*p* > 0.05) and 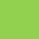 mean significant (*p* < 0.05) improvement were observed in the experimental group after plyometric jump training compared with the control group; ^a^ Data denote the number of included studies (total number of participants included). Abbreviations: SMD, standardized mean difference; CI, confidence interval; PT, plyometric training.

Eleven studies (n = 300) assessed the linear sprint performance of youth basketball players, revealing moderate heterogeneity among the studies (I^2^ = 61.4%, *p* < 0.001). Consequently, a random-effects model was applied. The results indicated a significant improvement in linear sprint performance (SMD = 0.59, 95% CI: 0.25 to 0.94, *p* < 0.001) (Supplementary Appendix S2; Supplementary Figure S3; Figure 3). A further breakdown of linear sprint performance showed that PT significantly enhanced the 5–10 m (*p* < 0.05) and 20–30 m (*p* < 0.01) sprint capabilities of youth basketball players.

Regarding participant characteristics: 1) PT significantly enhanced the linear sprint performance of male youth basketball players (*p* = 0.001), but no significant enhancement was observed for female players (*p* = 0.567); 2) PT significantly improved the linear sprint performance of basketball players aged 5 to 10.99 (*p* < 0.05) and 11–14.99 years (*p* < 0.05), while no significant improvements were found for players aged 15–17.99 years (*p* = 0.134). In terms of training protocols: 1) Both 6–8 weeks (*p* < 0.05) and >8 weeks (*p* = 0.001) of PT significantly improved the linear sprint performance of youth basketball players; 2) PT sessions ≤2 times per week (*p* < 0.001) significantly improved linear sprint performance, whereas >2 times per week (*p* = 0.056) did not show significant improvement; 3) PT with jumping repetitions >150 times per week (*p* = 0.001) significantly improved linear sprint performance, whereas ≤150 times per week (*p* = 0.157) did not show significant improvement; 4) Mixed-type PT improved linear sprint performance in youth basketball players (*p* = 0.001), whereas single-type PT did not result in significant improvements (*p* = 0.194).

#### 3.2.4 Effect of PT on COD speed

**FIGURE 4:**
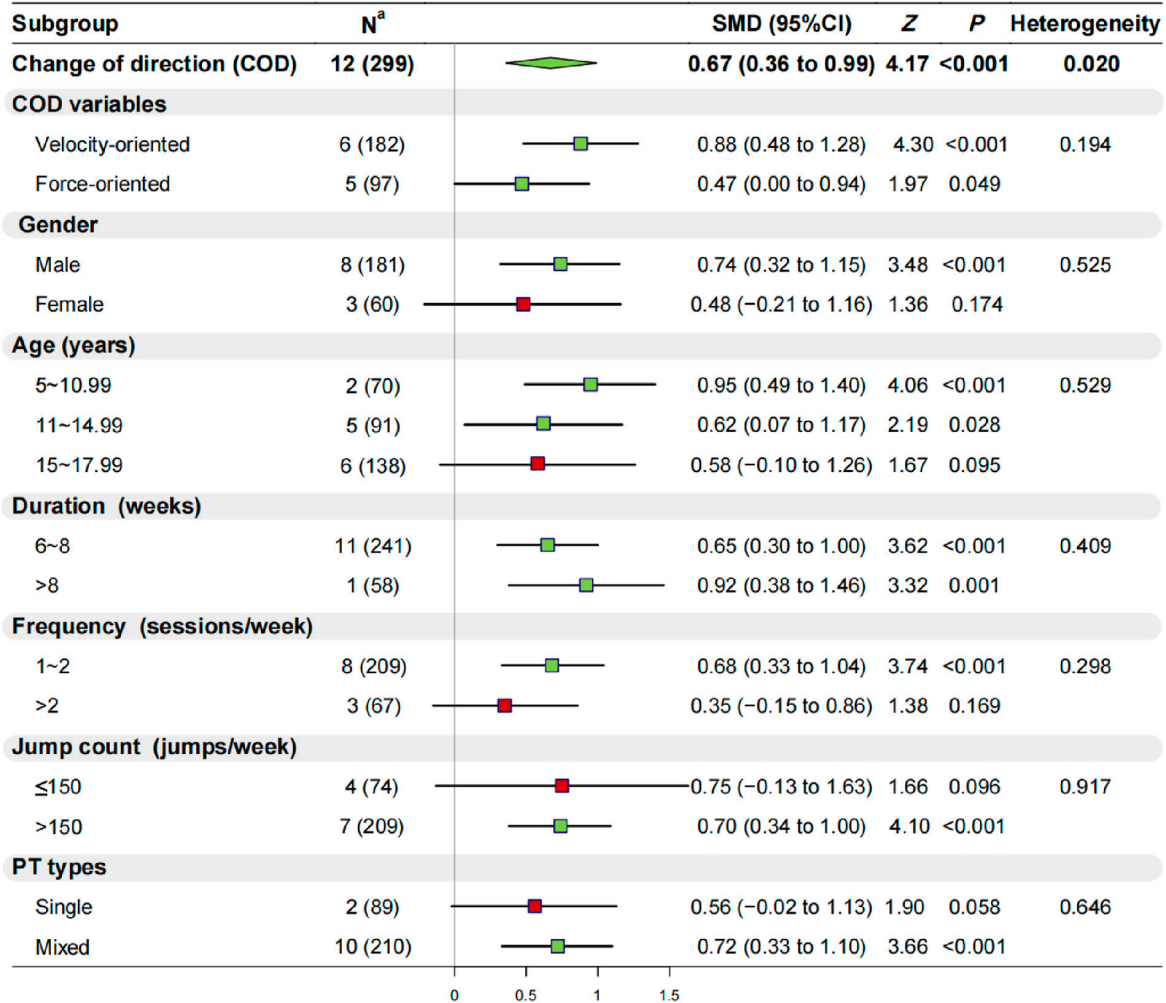
Results of Meta-Analysis and Subgroup Analysis on COD speed Note 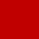 mean nonsignificant (*p* > 0.05) and 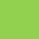 mean significant (*p* < 0.05) improvement were observed in the experimental group after plyometric jump training compared with the control group; ^a^ Data denote the number of included studies (total number of participants included). Abbreviations: COD, change of direction; SMD, standardized mean difference; CI, confidence interval; PT, plyometric training.

Twelve studies (n = 299) assessed the COD speed of youth basketball players, revealing moderate heterogeneity among the studies (I^2^ = 47.0%, *p* = 0.02), warranting a random-effects model for analysis. The results showed statistically significant differences (SMD = 0.67, 95% CI: 0.36 to 0.99, *p* < 0.001) (Supplementary Appendix S2; Supplementary Figure S4; Figure 4), indicating that PT significantly enhances the COD speed of youth basketball players. Further subdivision of COD speed revealed that PT significantly improved both velocity-oriented (*p* < 0.001) and force-oriented COD speed (*p* < 0.05).

Concerning participant characteristics: 1) PT significantly enhanced the COD speed of male youth basketball players (*p* < 0.001), but no significant enhancement was observed for female players (*p* = 0.174); 2) PT significantly enhanced the COD speed of basketball players aged 5–10.99 years (*p* < 0.001) and 11 to 14.99 (*p* < 0.05), while no significant improvements were found for players aged 15 to 17.99 (*p* = 0.095). Regarding training regimens: 1) Both 6–8 weeks (*p* < 0.001) and >8 weeks (*p* = 0.001) of PT significantly improved the COD speed of youth basketball players; 2) PT sessions ≤2 times per week (*p* < 0.001) significantly improved COD speed, whereas >2 times per week (*p* = 0.169) did not show significant improvement; 3) PT with jumping repetitions >150 times per week (*p* < 0.001) significantly improved COD speed, whereas ≤150 times per week (*p* = 0.096) did not show significant improvement; 4) mixed-type jump training significantly improved the COD speed of youth basketball players (*p* < 0.001), whereas single-type jump training did not show significant improvement (*p* = 0.058).

#### 3.2.5 Effect of PT on balance

**FIGURE 5:**
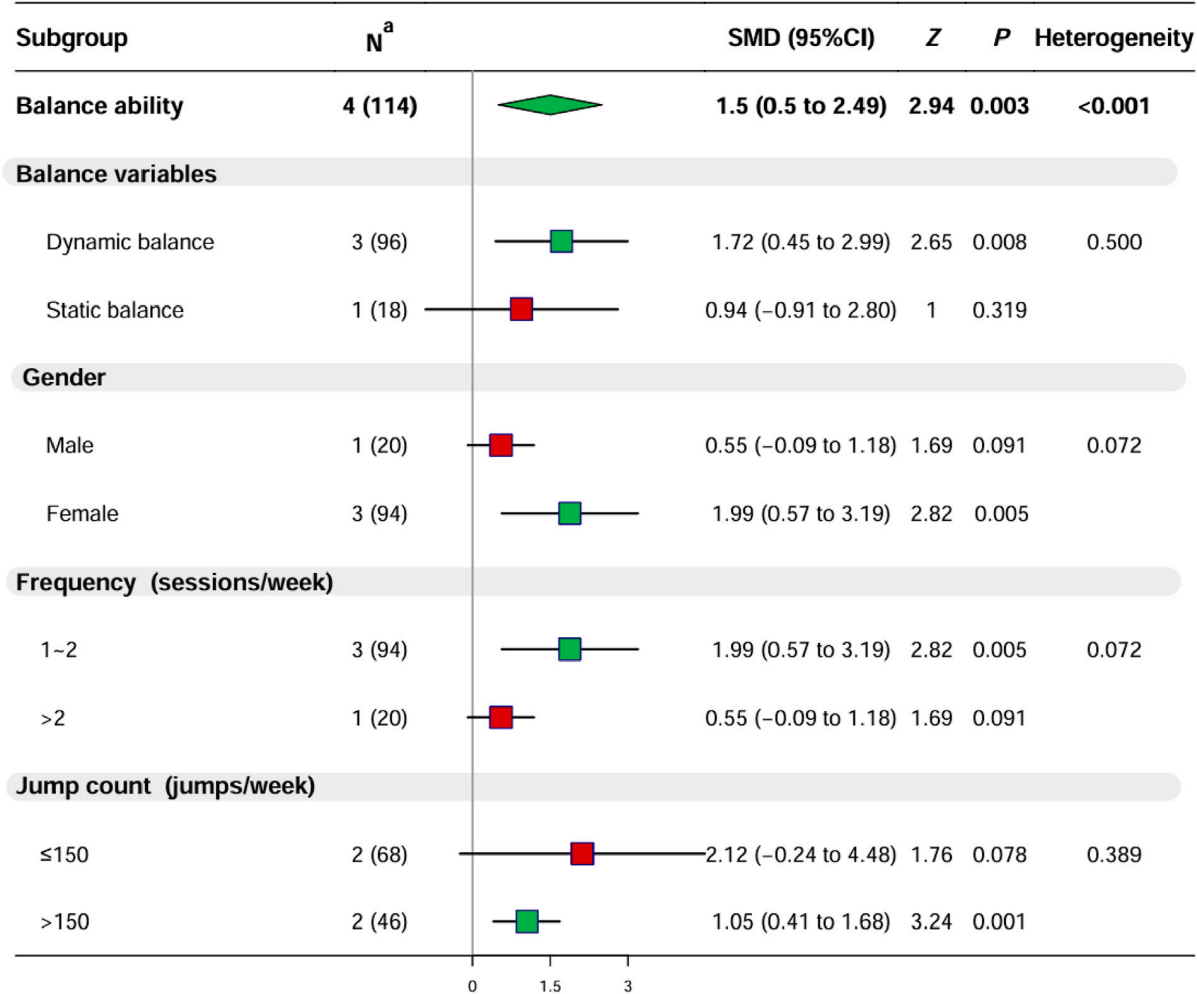
Results of Meta-Analysis and Subgroup Analysis for Balance Ability. Note: 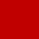 mean nonsignificant (*p* > 0.05) and 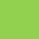 mean significant (*p* < 0.05) improvement were observed in the experimental group after plyometric jump training compared with the control group; ^a^ Data denote the number of included studies (total number of participants included). Abbreviations: SMD, standardized mean difference; CI, confidence interval; PT, plyometric training.

Four studies (n = 114) assessed the balance of youth basketball players, revealing high heterogeneity among the studies (I^2^ = 88.9%, *p* < 0.001), necessitating using a random-effects model for analysis. The results indicated that PT significantly enhanced the balance ability of youth basketball players (SMD = 1.50, 95% CI: 0.50 to 2.49, *p* < 0.001) (Supplementary Appendix S2; Supplementary Figure S5; Figure 5). Further differentiation of balance ability revealed that PT only significantly improved dynamic balance (*p* < 0.01), with no significant enhancement observed in static balance (*p* = 0.319).

Regarding participant characteristics, PT significantly improved the balance ability of female youth basketball players (*p* < 0.05), whereas the enhancement in balance ability for male participants was not significant (*p* = 0.091). In terms of training characteristics: 1) PT sessions ≤2 times per week (*p* < 0.01) significantly improved balance, whereas >2 times per week (*p* = 0.091) did not show significant improvement; 2) PT with jumping repetitions >150 times per week (*p* = 0.001) significantly improved balance ability, whereas ≤150 times per week (*p* = 0.078) did not show significant improvement.

### 3.3 Sensitivity analysis, publication bias analysis, and evidence quality assessment

**FIGURE 6:**
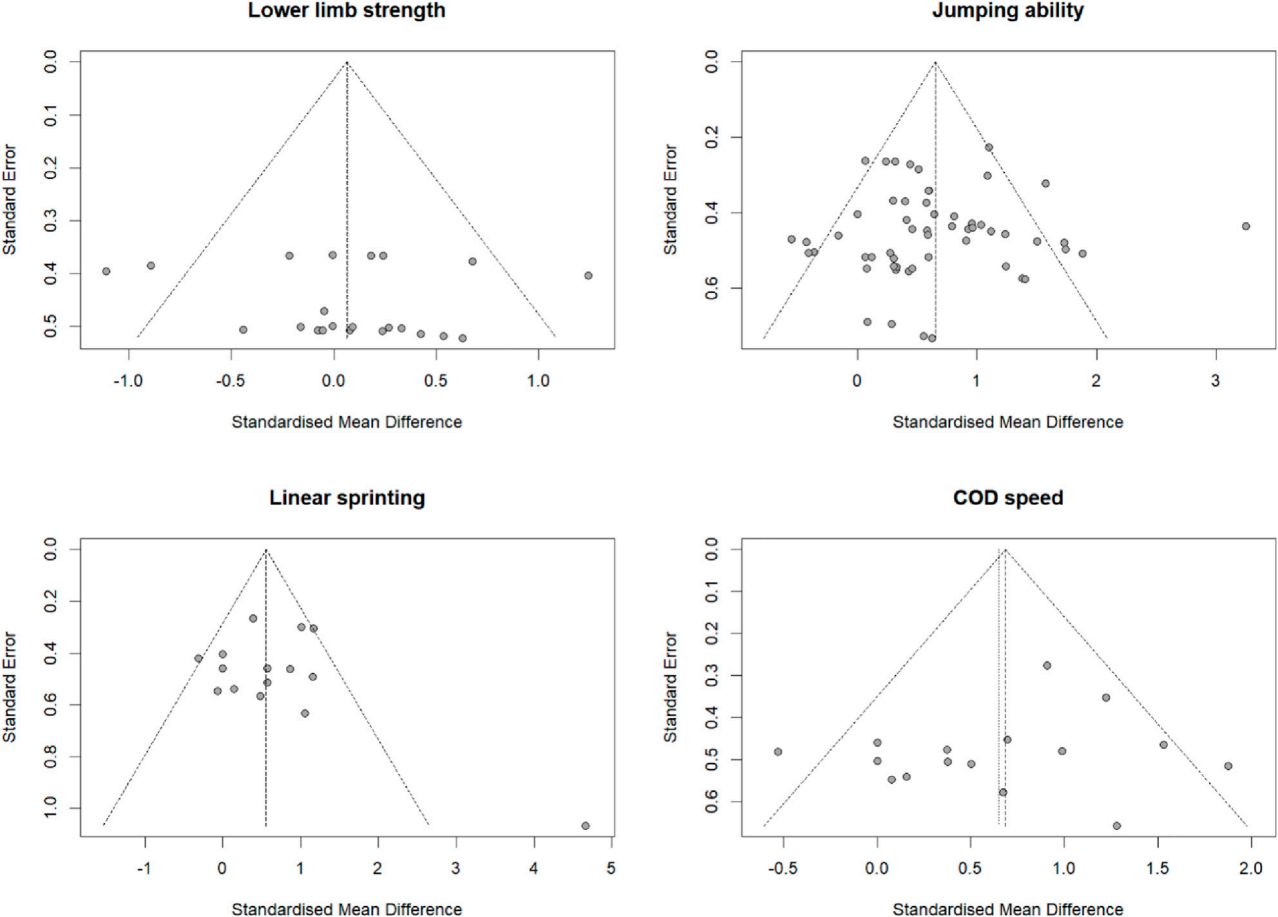
Funnel Plot for Publication Bias of the Included Studies Abbreviations: COD, Change of direction.

Sensitivity analyses were conducted on lower limb strength, jumping ability, COD speed, linear sprint, and balance using a sequential omission method. The results showed that the overall effect size for various athletic performance parameters remained essentially unchanged, indicating the high robustness of the meta-analysis results (Table 3). Egger’s test assessed publication bias for lower limb strength, jumping, linear sprint, COD speed, and balance ability, and funnel plots were created for performance measures involving more than ten studies. The results of Egger’s test were not statistically significant (*p* > 0.05) (Table 3), and the distribution of studies within the funnel plots was roughly symmetrical (Figure 6), suggesting an absence of evident publication bias.

**TABLE 3:** Sensitivity analysis, publication bias analysis, and GRADE assessment of the level of evidence.

Fitness attributes	Combined effect after excluding individual studies			Publication bias	GRADE
	SMD	P	I^2^	Egger’s	
Lower limb strength	0.00∼0.13	0.17∼0.97	7%∼36%	0.607	Low^1^ ^,^ ^3^
Jumping ability	0.64∼0.70	<0.001 ∼ <0.001	36%∼55%	0.909	Low^1^ ^,^ ^2^
Linear sprinting	0.52∼0.66	<0.001 ∼ <0.001	36%∼64%	0.413	Very low^1^ ^,^ ^2^ ^,^ ^3^
COD speed	0.60∼0.77	<0.001∼0.001	32%∼49%	0.396	Low^1^ ^,^ ^3^
Balance ability	0.97∼1.70	<0.001∼0.01	62%∼90%	0.526	Very low ^1^ ^,^ ^2^ ^,^ ^3^

Note: GRADE, Criteria for Downgrading Evidence Quality.

^1^ Risk of Bias.

^2^ Inconsistency.

^3^ Imprecision.

Abbreviations: SMD, standardized mean difference; COD, change of direction.

## 4 Discussion

This meta-analysis aimed to explore the effects of PT on the athletic performance of youth basketball players. The overall results of the study indicate that, compared to active controls, PT may generally improve athletes’ jumping, linear sprinting, COD speed, and balance abilities, with medium to large effect sizes. However, there was no significant improvement in lower limb strength. Subgroup analysis revealed that the effectiveness of PT is related to the participant’s age, with the 11–14.99 age range potentially being a critical period for basketball players to develop athletic performance through PT. Furthermore, this study found that 6–8 weeks of training is sufficient to improve the athletic performance of youth basketball players, and the effects of training further enhanced with extended training duration (>8 weeks). The most effective PT program involved low frequency (≤2 sessions per week), a high number of repetitions (>150 PT sessions), and a mixed-type of PT.

### 4.1 The effects of PT on different athletic performances

#### 4.1.1 Effect of PT on lower limb strength

Strength is fundamental to athletic performance, and superior lower limb strength contributes to basketball players’ enhanced speed, jumping, and balance abilities (Suchomel et al., 2016). However, this study’s results indicate that PT does not significantly enhance lower limb strength in youth, consistent with previous meta-analysis results on youth athletes (Oliver et al., 2024; Ramirez-Campillo et al., 2022). The analysis suggests that the studies included in this research utilized isokinetic dynamometers to assess lower limb strength. Isokinetic dynamometry is a safe and reliable method for evaluating the strength and power of muscle groups and is considered the “gold standard” for assessing open kinetic chain movements (Lourencin et al., 2012). However, isokinetic strength testing requires practitioners to contract muscles at a constant speed (Zapparoli and Riberto, 2016), whereas PT typically involves rapid jumps and high-intensity short-duration contraction training (Sole, 2017). Therefore, the rapid contraction forms in PT may differ from the force application forms in isokinetic dynamometer testing. Training effects are usually reflected in movements similar to the training mode (Randell et al., 2010), so isokinetic strength testing may not fully reflect the impact of PT on strength quality. Additionally, isokinetic dynamometry assesses the maximum strength output of specific muscle groups, such as the hamstrings and quadriceps (Zapparoli and Riberto, 2016), whereas PT mainly improves overall athletic performance by enhancing muscle coordination, relying on the coordinated work of multiple muscle groups, rather than enhancing the strength of a single muscle group (Sole, 2017), which may also result in non-significant strength test results in this study.

Although the optimal program for improving strength is not entirely clear, previous meta-analyses have indicated that combining PT with resistance training can enhance lower limb strength by approximately three times compared to PT alone (ES: 0.27 vs 0.75) (Oliver et al., 2024). The increase in muscle strength is believed to be stimulated by high-tension muscle fiber activation, which is why heavy loads are usually used to improve muscle strength (Rodríguez-Rosell et al., 2016). Accordingly, prior meta-analyses have shown that strength training with loads below 60%–80% of 1RM does not effectively increase strength (Kamper and Michaleff, 2011). Therefore, whether incorporating some resistance training into PT programs or conducting weighted PT is necessary to effectively enhance lower limb strength in youth basketball players requires further investigation.

#### 4.1.2 Effect of PT on jumping

In basketball, exceptional jumping ability can give athletes a significant advantage in rebounding and blocking opponents (Arede et al., 2019). This study found that PT can enhance the jumping ability of youth basketball players, consistent with findings from previous meta-analyses on soccer (Pardos-Mainer et al., 2021) and volleyball players (Ramirez-Campillo et al., 2020). Jumping ability is often used as a surrogate indicator of lower limb strength. However, our study revealed that PT improved jumping ability while showing no significant enhancement in isokinetic muscle strength tests. This discrepancy may arise from the different adaptive changes elicited by various training methods. Resistance training excels in increasing muscle mass and maximum strength, thereby enhancing baseline strength levels (the ability to generate greater force) (Whitehead et al., 2018). In contrast, PT is more effective in power gains and rapid strength improvements, optimizing the conversion of baseline strength into athletic performance (completing movements more quickly) (McKinlay et al., 2018; Chaouachi et al., 2014). Improvements in jumping performance with PT may be attributed to various adaptive mechanisms, such as enhanced motor unit recruitment, greater inter-muscular coordination, heightened neural drive to agonist muscles, and enhanced utilization of the SSC” (Ramirez-Campillo et al., 2022). Additionally, other factors such as the transformation to type II muscle fibers, increased muscle contraction amplitude, and changes in muscle pennation angle may also contribute (Markovic and Mikulic, 2010). These adaptations help athletes more fully utilize muscle strength in actual gameplay, thereby improving jumping ability.

#### 4.1.3 The effect of PT on linear sprinting ability

Superior linear sprinting ability is instrumental in enabling basketball athletes to rapidly initiate offense and defense, significantly enhancing their efficiency (Wen et al., 2018). This study found that PT can enhance the linear sprinting ability of youth basketball players, which is consistent with the results of a previous meta-analysis on soccer players (Pardos-Mainer et al., 2021; Ramirez-Campillo et al., 2020). Firstly, PT can increase the level of neuromuscular activation in the trained muscles, as evidenced by the higher recruitment of motor units—primarily type II fibers—and enhanced synchronization levels (Häkkukinen et al., 1985).

These changes are conducive to the enhancement of maximal muscle strength, allowing athletes to achieve greater acceleration in the early phases of sprinting, and maintain higher speeds and longer stride length throughout the sprint (Morin et al., 2012; Bishop and Girard, 2013). Secondly, PT can induce changes in neuromechanical adaptability in the lower limb muscles and tendons, manifested as increased neural activation of the agonist muscles and enhanced elasticity of muscles and tendons (Markovic and Mikulic, 2010; Radnor et al., 2018). These changes are beneficial for enhancing the efficacy of the SSC, thereby enabling the concentric phase to generate greater force and improve sprint performance. It is important to note the strong correlation between jumping ability and linear sprinting ability, with horizontal jumping being particularly crucial during the acceleration phase of a sprint (≤10 m) and vertical ground forces becoming more prominent as speed increases beyond this point (>10 m) (Morin et al., 2012; Ramirez-Campillo et al., 2015). Therefore, incorporating both horizontal and vertical jumps in PT may be an appropriate strategy for improving sprint performance in basketball players.

#### 4.1.4 The effect of PT on COD speed

Enhancing COD speed allows athletes to change direction and modify actions to outmaneuver opponents rapidly. This study found that PT can increase COD speed in youth basketball players, consistent with the results of previous meta-analyses of childhood adolescents (Asadi et al., 2015) and female soccer players (Pardos-Mainer et al., 2021). COD speed involves braking with eccentric muscle contractions followed by concentric contractions to provide propulsion (Castillo-Rodriguez et al., 2012). This requires applying a significant amount of force to the ground within a short time frame. According to COD speed tests, maximum force production takes approximately 0.44–0.72 s (DeWeese and Nimphius, 2016), indicating that exercises aimed at improving COD speed should focus on exerting maximum force within this time frame. Reaction intensity is a subcategory affecting COD speed (Young et al., 2002), referring to the ability to transition as quickly as possible from eccentric to concentric muscle actions in the SSC, as seen in CMJ, DJ, and COD speed. PT aims to apply a large amount of force in a short time, targeting increased power output, which is determined by the force and speed involved in SSC (Potach and Chu, 2016). The similarity between COD speed actions and SSC suggests that PT can enhance COD speed (Asadi et al., 2015; Pardos-Mainer et al., 2021). Furthermore, PT improves neuromuscular coordination and proprioceptive function, enhancing the interaction of major muscle groups during movement, including the improved capabilities of active lower limb muscles, synergists, and antagonists in eccentric braking and rapid eccentric-concentric transitions, which leads to better muscle coordination, improved movement continuity, and enhanced force transmission, ultimately improving COD speed (Sheppard and Young, 2006; Young and Farrow, 2006; Young et al., 2015).

#### 4.1.5 The effect of PT on balance ability

Good balance enhances body stability in basketball players and reduces the risk of lower limb injuries (Ramirez-Campillo et al., 2022). Our study finds that PT improves balance in youth basketball players, which is consistent with the results of a previous meta-analysis of all-age basketball (Ramirez-Campillo et al., 2022). PT is a dynamic training method involving rapid SSC and vertical and horizontal shifts in the center of gravity (Komi and Bosco, 1978). This training improves proprioceptive function and enhances the athlete’s ability to control their center of gravity (Asadi et al., 2015). Additionally, PT significantly improves neuromuscular control by promoting anticipatory postural adjustments (Gantchev and Dimitrova, 1996). Previous research indicates that anticipatory postural adjustments primarily involve peripheral joints. In jump training, repeated exposure to challenges in balance and stability encourages the body to make active or feedforward adjustments, preparing the muscles before landing (Marigold and Patla, 2002; Paillard et al., 2005). PT can also enhance the sensitivity of afferent feedback pathways (Borghuis et al., 2008).

Moreover, anticipatory or feedforward adjustments contribute to injury prevention (Chimera et al., 2004), which is crucial for basketball players. Compared to other sports, basketball players have the highest incidence of ACL injuries (RR = 4.14), with seasonal risks of 1.03% for girls and 0.25% for boys (Bram et al., 2021). Previous studies have indicated that PT can reduce the incidence of ACL injuries in athletes (Markovic and Mikulic, 2010; Wilkerson et al., 2004). Although this study did not directly measure these outcomes, it suggests that PT not only enhances the athletic performance of adolescent basketball players but also plays a critical role in injury prevention and extending athletic careers.

#### 4.1.6 Subgroup analysis of athletic performance

To further explore the effects of PT on various aspects of athletic performance, a subgroup analysis was conducted. The results indicate that PT enhances vertical jump, sprinting ability over 5–10 m and 20–30 m, velocity-oriented and force-oriented COD speed, and dynamic balance in youth basketball players. However, there were no significant improvements in horizontal jumping and static balance.

These findings align with the principle of training specificity, which supports using vertical and horizontal jumps to better improve performance in vertical and horizontal directional tasks (Randell et al., 2010). A meta-analysis by Moran et al. (2021) also shows that horizontal PT significantly improves horizontal jump performance compared to vertical PT (ES: 0.65), while having similar effects on vertical jumps as vertical PT. Therefore, horizontal PT may be a more effective method for improving multi-directional movement performance. In basketball, where vertical jumping is predominant, this is a notable difference from sports like sprinting and long jumping. Most studies included in this research employed vertical PT, which may have contributed to the lack of significant improvement in horizontal jump tests. Based on these results, it seems logical to recommend that coaches incorporate a higher proportion of horizontal PT into their training programs, as horizontal movement performance is also crucial for basketball players (Moran et al., 2021).

Regarding static balance, our results are inconsistent with previous studies, as a meta-analysis on healthy populations indicated that PT significantly improved both dynamic and static balance (Ramachandran et al., 2021). However, only one study in the static balance subgroup, conducted by Meszler and Váczi (2019), employed a 7-week training program. This program included daily weekday training sessions and one to two basketball games on weekends, potentially limiting athletes’ recovery time from fatigue. Fatigue comprises central and peripheral components (McKenna and Hargreaves, 2008), and is characterized by reduced muscle activation, decreased motor neuron firing frequency and synchronization, diminished motor cortex drive, decreased muscle fiber contraction strength, and altered muscle action potential transmission mechanisms (Tornero-Aguilera et al., 2022; Zając et al., 2015). Due to the limited number of studies that have tested balance abilities (n = 4), however, these findings should be approached with caution, and the effectiveness of PT on static balance in youth basketball players requires further investigation with more studies.

### 4.2 Effects of plyometric training on different subjects

The study demonstrates that PT can enhance specific aspects of athletic performance in youth. However, as a unique demographic, youth may exhibit varying responses to PT due to differences in their physical and psychological developmental stages. Consequently, this study stratified the subjects by age and gender to elucidate the performance changes across different age groups and genders following PT. This stratification aims to provide a theoretical foundation for developing more personalized training programs by identifying the critical periods of growth and development in youth.

#### 4.2.1 Age

In this study, subjects were divided into three age groups: 5–10.99 years, 11–14.99 years, and 15–17.99 years. Due to the concentration of balance ability in the 15 to 17.99 age group, only jump, linear sprint, and COD speed were analyzed in the subgroups. The results indicated that only basketball players aged 11 to 14.99 showed significant improvements in jump, linear sprint, and COD speed, with effect sizes reaching medium to large levels (ES: 0.94, 0.90, 0.62, respectively). For jump and linear sprint, the effect size was greatest in the 15 to 17.99 age group compared to other ages, while in COD speed, the 5 to 10.99 age group showed a greater improvement than the 11 to 14.99 age group (ES: 0.95 vs 0.62).

According to the YPD model Lloyd and Oliver, 2012, ages 12 to 16 for males and 11 to 15 for females represent critical periods for athlete development, during which agility, speed, power, strength, and endurance are highly sensitive to training. During this time, testosterone and growth hormone levels increase rapidly (Fragala et al., 2011; Malina et al., 2004), promoting muscle and strength development (Boisseau and Delamarche, 2000; Granacher et al., 2011). Consequently, athletes in this age range can better leverage these physiological changes to significantly enhance their jump, linear sprint, and COD speed. In terms of COD speed, the 5 to 10.99 age group had a higher effect size than the 11 to 14.99 age group (ES: 0.95 vs 0.62). Pre-puberty is a time frame where children undergo neuro-coordination and central nervous system maturation (Myer et al., 2013; Sowell et al., 2004), with brain maturity peaking between 6 to 8 years and 10–12 years. It can be posited that the high neural demands of PT provide a stimulus that aligns with the natural adaptive responses from growth and maturation in pre-peak Height Velocity (Lloyd and Oliver, 2012), consistent with the YPD model Lloyd and Oliver, 2012. These findings may reflect a process of “synergistic adaptation” which denotes a symbiotic relationship between specific adaptations to imposed training demands and concurrent growth and maturity-related adaptations. This synergistic relationship likely leads to amplified training responses related to age. Additionally, Previous research on the peak improvement window for sprinting ability in adolescent soccer players suggests that this window may occur at 13.8 ± 0.8 years of age, though some adolescents achieve their peak speed improvement before this window begins (Philippaerts et al., 2006). these findings underscore the necessity of age-specific PT while also considering the individual growth and development characteristics of athletes to fully capitalize on the physiological advantages inherent in each age group, thereby achieving optimal enhancements in athletic performance.

#### 4.2.2 Sex

This study found that youth exhibit gender-based differences in their adaptability to PT. The results indicate that PT enhances jumping ability in both male and female youth, with additional improvements in speed and COD speed for males and balance for females. This contrasts with previous studies, which found no gender differences in training adaptability for adults in aspects such as sprinting, COD speed (Ramírez-Campillo et al., 2016) and balance (Ramachandran et al., 2021). However, during the period of rapid growth and development, youth exhibit physiological gender differences that distinguish them from adults. Prior to puberty, the development rates of strength, speed, explosive power, endurance, and coordination are similar between boys and girls (Beunen and Malina, 2008). After the onset of puberty growth spurts, almost all physical attributes exhibit significant maturity differences, with males generally showing greater improvements in most physical qualities except flexibility (Malina et al., 2004; Beunen and Malina, 2008). Typically, girls experience their puberty growth spurt approximately 2 years earlier than boys (around age 10 for girls and age 12 for boys) (Beunen and Malina, 2008), but boys’ growth spurts are more pronounced (Beunen and Malina, 1988). The YPD model also suggests differences in the adaptive periods for physical attributes between males and females Lloyd and Oliver, 2012. These differences could lead to gender-based variations in PT adaptability during puberty. However, due to the limited number of studies included in this analysis, further subdivision by gender within each age group was not possible. Additionally, the included studies had an unbalanced gender ratio, with females comprising only 25.04% of the sample. This imbalance might explain why females, despite having a similar effect size to males in COD speed (ES: 0.48 vs 0.74), did not achieve statistical significance, likely due to the significantly smaller sample size for females (n = 60) compared to males (n = 161). Similarly, for balance tests, only one study with male participants (n = 20) was included, producing a moderate effect size (ES: 0.55) but no significant difference compared to the active control group. Therefore, these results should be interpreted with caution. Future research with more balanced and extensive data is necessary to validate the differences in PT impacts between male and female youth.

### 4.3 The impact of different training protocols on PT

This meta-analysis compared the changes in various athletic performances observed in the included studies. Differences in participant characteristics may help explain the optimal training age for youth. Similarly, variations in PT training protocols (such as training weeks, weekly frequency, PT sessions per week, and PT types) could also contribute to the differing degrees of performance improvement reported across studies. To analyze this possibility, the impact of potential moderating variables was explored. Investigating the differences in training protocols between studies aims to provide a theoretical basis for the development of future PT training programs.

#### 4.3.1 Training duration

The studies included in this meta-analysis were categorized based on the duration of PT training: 6–8 weeks and >8 weeks. As the studies focusing on balance abilities had a maximum duration of 8 weeks, the effects of interventions lasting more than 8 weeks could not be examined for balance. Subgroup analysis revealed that PT training for 6–8 weeks could improve jump, sprint, and COD speed in youth basketball players, with moderate effect sizes (ES: 0.60, 0.52, 0.65, respectively). However, training periods longer than 8 weeks resulted in more significant improvements, with large effect sizes (ES: 0.81, 0.85, 0.92, respectively). This suggests that while 6–8 weeks of PT training is sufficient to enhance these abilities, extending the training duration can yield greater benefits. Therefore, PT could be effectively integrated into long-term basketball training programs for youth.

#### 4.3.2 Training frequency

Subgroup analysis based on intervention frequency found that PT training >2 times per week significantly improved jump ability but did not significantly enhance speed, COD speed, and balance. In contrast, training ≤2 times per week significantly improved jump, speed, COD speed, and balance abilities in youth basketball players, with moderate to large effect sizes (ES: 0.76, 0.59, 0.68, 1.99, respectively). This indicates that lower-frequency PT training can provide greater benefits, aligning with previous meta-analysis results in soccer (Pardos-Mainer et al., 2021; Ramirez-Campillo et al., 2020; de Villarreal et al., 2009).

Training frequency should be considered in terms of recovery and efficiency. Although the mechanisms underlying the effectiveness of lower frequency PT training are not entirely clear, it may be related to athlete recovery and adaptation. PT places high demands on the neuromuscular system and is a high-intensity training modality. PT can lead to acute fatigue of SSC functions, and the fatigue-recovery process often exhibits a bimodal trend, with a short recovery phase followed by a performance decline lasting 2–3 days (Miller et al., 2002). In the included studies, athletes also engaged in regular basketball training and competitions. If PT training exceeds three times per week, athletes may not have sufficient recovery time, hindering optimal recovery and adaptation (Peake, 2019). In terms of efficiency, Javier et al. (2017) explored the effects of 1–2 PT sessions per week on the performance of 24–years–old male futsal players, finding that weekly PT sessions resulted in better performance improvements than bi-weekly sessions. This suggests the efficacy of lower-frequency PT training. From a practical perspective, lower PT training frequencies allow athletes to allocate more time to other critical aspects of their preparation (e.g., shooting and tactical coordination) (Bouguezzi et al., 2020). This finding suggests that coaches need not prioritize excessively high training frequencies when designing training programs.

#### 4.3.3 Weekly training volume

The results of this study indicate that for youth basketball players, only PT involving >150 jumps per week can significantly enhance linear sprint and COD speed. Previous meta-analyses on the effects of PT on a broader age range (including both adults and adolescents) have shown that training sessions with >80 jumps per session, conducted 3–4 times per week (>240–320 jumps per week) over a period of 6–8 weeks, and totaling >18 sessions may be most effective in improving sprint performance (de Villarreal et al., 2012). Another similar meta-analysis suggested that training lasting >10 weeks, with >20 sessions and >50 high-intensity jumps per session (>100 jumps per week), seems to maximally enhance overall athletic performance (de Villarreal et al., 2009). These findings suggest that there may be a dose-response threshold for the effectiveness of PT.

However, specific interventions in this study revealed that the number of weekly training sessions was not always consistent. For example, some studies reported an average of >150 jumps per week (averaging 187, 181, 199, and 378 jumps per week) over 6–8 weeks, employing a progressive training structure with bi-weekly increments in training volume (Sidki Adigüzel and Günay, 2016; Bouteraa et al., 2020; Cigerci and Genc, 2020). Other studies combined “progressive training” with “constant training,” maintaining a gradual increase in jumps per week for the first 8 weeks and then keeping the same number of jumps in weeks 9 and 10 (Latorre-Román et al., 2017). Some employed a combination of “progressive training” and “regressive training,” with a gradual increase in jumps per week for the first 6 weeks, followed by a reduction to the week 1 level in the last week (Hernández et al., 2018). Additionally, some studies combined “constant load” with “regressive training” (averaging 166 jumps per week) (Santos and Janeira, 2008) or used “non-linear periodization” (averaging 166 jumps per week) (Santos and Janeira, 2011).

Furthermore, the different intensity levels resulting from various jumping exercises in PT, such as unilateral and bilateral jumps with body weight as the load, depth jumps (at different heights), CMJ, and alternating leg jumps, can also influence the dose-response effect on performance (Andrade et al., 2020). This suggests that, although this study established the beneficial effect of weekly PT jump counts on the sprinting ability of youth basketball players, the precise dose-response relationship between specific jump counts and sprinting ability requires further investigation in future studies. In conclusion, although the dose-response relationship is not clearly defined, there is a strong positive correlation between total jump counts and athletic performance in sprinting and COD speed.

#### 4.3.4 Types of PT

Based on the number of PT types utilized in the training program, PT was divided into single-type PT and mixed-type PT. The results indicated that single-type PT could only enhance the jumping ability of adolescent basketball players, whereas mixed-type PT could simultaneously improve jumping, linear sprinting, and COD speed, with medium effect sizes. Moreover, the improvements in jumping, linear sprinting, and COD speed were superior to those achieved by single-type PT (ES: 0.74 vs 0.45; 0.70 vs 0.28; 0.72 vs 0.56). The analysis suggests that mixed-type PT includes various types of PT training, such as lateral jumps, box jumps, and single-leg hurdle hops. Different types of jumps can induce specific neuromuscular adaptations (Lloyd et al., 2016; Ramírez-Campillo et al., 2015). For instance, CMJ is advantageous for developing agility, while DJ is more effective for increasing vertical jump height (Thomas et al., 2009). Horizontal PT tends to enhance short-distance (≤10 m) acceleration, whereas vertical PT is inclined to improve longer-distance (10–20 m) top speed (Loturco et al., 2015; Ramírez-Campillo et al., 2015). Unilateral PT can rapidly increase an athlete’s strength qualities, while bilateral PT offers longer-lasting performance gains (Makaruk et al., 2011). Compared to the relatively fixed exercises and stimuli of single-type PT, mixed-type PT can bring about more comprehensive improvements in athletic abilities. This suggests that coaches should incorporate various PT types into training programs to more effectively enhance the athletic performance of adolescent basketball players.

## 5 Limitations of the study

This research has certain limitations that should be acknowledged. 1) The quality of evidence assessed using the GRADE system was low to very low. This downgrading was primarily due to the lack of blinding in the studies, high heterogeneity in some research, and insufficient sample sizes; 2) Some subgroup analyses were based on only one or two studies, so the results from these subgroups should be interpreted with caution; 3) The included studies utilized various measurement tools, which may differ in their validity and reliability, potentially affecting the consistency of the results; 4) Due to limitations in the data available from the literature, not all relevant outcome measures of athletic performance could be comprehensively included. Consequently, the impact of PT on a broader range of sports performance outcomes (such as endurance, scoring ability, shooting accuracy, etc.) requires further investigation.

## 6 Conclusions and recommendations

1) PT can enhance the jump ability, straight sprinting ability, COD speed, and balance in youth basketball players, but it does not appear to improve lower limb strength. 2) Regarding the effect of PT on different aspects of athletic performance, enhancements were found for vertical jump, 5–10 m, 20–30 m sprinting ability, velocity-oriented and force-oriented COD speed, and dynamic balance ability of youth basketball players. 3) When analyzing different participant subgroups, basketball players aged 5 to 10.99 and 11–14.99 years appeared to improve their jump, sprinting ability, and COD speed through PT training, whereas no improvements in sprinting ability and COD speed were found for players aged 15 to 17.99. Male and female youth basketball players could improve their jumping through PT. In contrast, straight-line sprinting ability and COD speed were significantly improved only by male youth basketball players, and balance ability was significantly improved only by female youth basketball players. 4) In terms of different training schemes, >2 times/week, ≤150 times/week and single-type PT only improved the jumping ability, while 1–2 times/week, >150 jumps/week, and mixed-type PT can significantly enhance jump ability, COD speed, straight sprinting ability, and balance, providing the most comprehensive improvement.

In conclusion, to enhance athletic performance in youth basketball players, coaches should not only consider the characteristics of the training program but also take into account the dynamic physiological changes during adolescence. Tailoring training programs to the age and gender characteristics of youth, capturing critical growth periods, integrating low-frequency, high-volume, and mixed-type PT into their daily training, and regularly assessing its effectiveness are essential. Furthermore, as no studies have yet reported injury events related to PT, future researchers are encouraged to document not only the benefits but also any pain or adverse reactions associated with PT to fully understand its effects and risks, ensuring a safer and more effective training regimen for athletes.

.
