# Supplementary material for: Meta-analysis of the effect of plyometric training on the athletic performance of youth basketball players
Source: Front Physiol. 2024 Sep 20;15:1427291. doi: 10.3389/fphys.2024.1427291 (PMC11457583; doi:10.3389/fphys.2024.1427291)
Supplement: Supplementary file 1 [file DataSheet1.docx]

| Reviewer 1 | Reviewer 2 | | | Total |
| --- | --- | --- | --- | --- |
|  | Exclude | Include | Unclear |  |
| Exclude | 728 | 2 | 11 | 741 |
| Include | 1 | 35 | 4 | 40 |
| Unclear | 3 | 3 | 15 | 21 |
| Total | 732 | 40 | 30 | 802 |

**Table S1** The inter-rater agreement for reading the title and abstract

Kappa score: 0.805 (0.733~0.874)

**Table S2** The inter-rater agreement for full-text review

| Reviewer 1 | Reviewer 2 | | | Total |
| --- | --- | --- | --- | --- |
|  | Exclude | Include | Unclear |  |
| Exclude | 46 | 1 | 2 | 49 |
| Include | 1 | 21 | 1 | 23 |
| Unclear | 0 | 1 | 2 | 3 |
| Total | 47 | 23 | 5 | 75 |

Kappa score: 0.829 (0.711~0.947)

| **Table S3** Physiotherapy Evidence Database (PEDro) scale ratings for the included studies. | | | | | | | | | | | | |
| --- | --- | --- | --- | --- | --- | --- | --- | --- | --- | --- | --- | --- |
| Study | Item number | | | | | | | | | | | Score |
|  | 1 | 2 | 3 | 4 | 5 | 6 | 7 | 8 | 9 | 10 | 11 |  |
| Adigüzel and Günay (2016) | 1 | 0 | 0 | 1 | 0 | 0 | 0 | 1 | 0 | 1 | 1 | 4/10 |
| Amato et al. (2018) | 1 | 1 | 0 | 1 | 0 | 0 | 0 | 0 | 0 | 1 | 1 | 4/10 |
| Andrejic (2012) | 1 | 1 | 0 | 1 | 0 | 0 | 0 | 1 | 1 | 1 | 1 | 6/10 |
| Arede et al. (2019) | 1 | 0 | 0 | 1 | 0 | 0 | 0 | 0 | 0 | 1 | 1 | 3/10 |
| Attene et al. (2015) | 1 | 1 | 1 | 1 | 0 | 0 | 0 | 1 | 1 | 1 | 1 | 7/10 |
| Aztarain-Cardiel et al. (2024) | 1 | 1 | 0 | 1 | 0 | 0 | 0 | 0 | 0 | 1 | 1 | 5/10 |
| Bouteraa et al. (2020) | 1 | 1 | 0 | 1 | 0 | 0 | 0 | 1 | 0 | 1 | 1 | 5/10 |
| Brown et al. (1986) | 1 | 1 | 0 | 1 | 0 | 0 | 0 | 1 | 0 | 1 | 1 | 5/10 |
| Chouhan et al. (2022) | 1 | 1 | 0 | 1 | 0 | 0 | 0 | 1 | 0 | 1 | 1 | 5/10 |
| Cigerci et al. (2020) | 1 | 0 | 0 | 1 | 0 | 0 | 0 | 1 | 0 | 1 | 1 | 4/10 |
| Fachina et al. (2017) | 1 | 1 | 1 | 1 | 0 | 0 | 0 | 1 | 0 | 1 | 1 | 6/10 |
| Fontenay et al. (2013) | 1 | 0 | 0 | 1 | 0 | 0 | 0 | 0 | 0 | 1 | 1 | 3/10 |
| Eliakim et al. (2014) | 1 | 1 | 0 | 1 | 0 | 0 | 0 | 1 | 0 | 1 | 1 | 5/10 |
| Haghighi et al. (2023) | 1 | 1 | 1 | 1 | 0 | 0 | 0 | 1 | 0 | 1 | 1 | 7/10 |
| Hernández et al. (2018) | 1 | 1 | 1 | 1 | 0 | 0 | 1 | 1 | 1 | 1 | 1 | 8/10 |
| Latorre-Román et al. (2018) | 1 | 1 | 0 | 1 | 0 | 0 | 0 | 1 | 0 | 1 | 1 | 5/10 |
| Matavulj et al. (2001) | 1 | 1 | 0 | 1 | 0 | 0 | 0 | 0 | 0 | 1 | 1 | 4/10 |
| McLeod et al. (2009) | 1 | 0 | 0 | 1 | 0 | 0 | 0 | 0 | 1 | 1 | 1 | 4/10 |
| Meszler and Váczi (2019) | 1 | 1 | 0 | 1 | 0 | 0 | 0 | 0 | 0 | 1 | 1 | 4/10 |
| Pamuk et al. (2022) | 1 | 1 | 0 | 1 | 0 | 0 | 0 | 0 | 0 | 1 | 1 | 5/10 |
| Santos and Janeira (2011) | 1 | 1 | 0 | 1 | 0 | 0 | 0 | 0 | 0 | 1 | 1 | 4/10 |
| Santos and Janeira (2009) | 1 | 1 | 0 | 0 | 0 | 0 | 0 | 1 | 0 | 1 | 1 | 4/10 |
| Santos and Janeira (2008) | 1 | 1 | 0 | 1 | 0 | 0 | 0 | 1 | 0 | 1 | 1 | 5/10 |
| Zribi et al. (2014) | 1 | 1 | 0 | 1 | 0 | 0 | 0 | 0 | 1 | 1 | 1 | 5/10 |
| Note: Item 1, eligibility criteria; Item 2, random allocation; Item 3, concealed allocation; Item 4, baseline comparability; Item 5, blind of subjects; Item 6, blind of therapists; Item 7, blind of assessors; Item 8, adequate follow-up; Item 9, intention-to-treat analysis; Item 10, between-group comparisons; Item 11, estimates and variability. | | | | | | | | | | | | |

**Table S4** The mean ± standard deviation (SD) of fitness variables were reported for both the plyometric jump training and control conditions in the studies included (Lower limb strength).

| Reference | Test | Experimental  (pre-test) | | Experimental  (pos-test) | | n | Control  (pre-test) | | Control  (pos-test) | | n |
| --- | --- | --- | --- | --- | --- | --- | --- | --- | --- | --- | --- |
|  |  | Mean | SD | Mean | SD |  | Mean | SD | Mean | SD |  |
| Adigüzel and Günay (2016) | 60°Right (Hamstring)Peak Power | 144.7 | 30.05 | 164.91 | 31.73 | 15 | 22.18 | 168.34 | 34.01 | 15 | 22.18 |
| Adigüzel and Günay (2016) | 60°Left (Hamstring)Peak Power | 123.12 | 20.98 | 125.36 | 26.12 | 15 | 16.24 | 129.76 | 24.41 | 15 | 16.24 |
| Adigüzel and Günay (2016) | 60°Right (Quadricep) Peak Power | 244.24 | 49.7 | 212.5 | 18.97 | 15 | 46.1 | 246.15 | 45.37 | 15 | 46.1 |
| Adigüzel and Günay (2016) | 60°Left (Quadricep) Peak Power | 212.5 | 18.97 | 183.68 | 23.09 | 15 | 23.14 | 191.69 | 16.84 | 15 | 23.14 |
| Adigüzel and Günay (2016) | 180°Right (Hamstring)Peak Power | 139.72 | 26.09 | 154.64 | 28.22 | 15 | 26.49 | 153.73 | 30.98 | 15 | 26.49 |
| Adigüzel and Günay (2016) | 180°Left (Hamstring) Peak Power | 122.85 | 19.06 | 132.63 | 21.91 | 15 | 14.36 | 127.06 | 21.04 | 15 | 14.36 |
| Adigüzel and Günay (2016) | 180°Right (Quadricep) Peak Power | 206.09 | 30.15 | 238.29 | 61.22 | 15 | 14.36 | 123.06 | 21.04 | 15 | 14.36 |
| Adigüzel and Günay (2016) | 180°Left (Quadricep) Peak Power | 193.67 | 24.67 | 187.6 | 36.92 | 15 | 14.36 | 123.06 | 21.04 | 15 | 14.36 |
| Matavulj et al. (2001) | Knee extensor strength, isometric (N·kg-1) | 7.3 | 1.9 | 6.6 | 1.7 | 11 | 1.3 | 6.5 | 1.7 | 11 | 1.3 |
| Matavulj et al. (2001) | Knee extensor strength, isometric (N·kg-1) | 7.2 | 1.7 | 6.7 | 1.3 | 11 | 1.3 | 6.5 | 1.7 | 11 | 1.3 |
| Meszler and Váczi (2019) | Knee extensor strength, dynamic 60°/s-1 (N.m-1) | 94.5 | 14.1 | 99.1 | 18 | 9 | 19.9 | 94.2 | 17.1 | 9 | 19.9 |
| Pamuk et al. (2020) | Isokinetic peak moment results of the knee during flexion and extension at 60°/s - Right (Nm) | 189.45 | 32.56 | 194.73 | 38.42 | 11 | 33.44 | 200.17 | 27.83 | 12 | 33.44 |
| Pamuk et al. (2020) | Isokinetic peak moment results of the knee during flexion and extension at 60°/s - Left (Nm) | 175.91 | 38.47 | 190 | 33.68 | 11 | 27.29 | 184.42 | 28.37 | 12 | 27.29 |
| Pamuk et al. (2020) | Isokinetic peak moment results of the knee during flexion and extension at 180°/s - Right (Nm) | 122.27 | 23.58 | 132.64 | 26.96 | 11 | 18.17 | 133.6 | 14.22 | 12 | 18.17 |
| Pamuk et al. (2020) | Isokinetic peak moment results of the knee during flexion and extension at 180°/s - Left (Nm) | 117.91 | 21.94 | 130 | 22.57 | 11 | 16.45 | 122.4 | 17.15 | 12 | 16.45 |
| Pamuk et al. (2020) | Isokinetic peak moment results of the knee during flexion and extension at 300°/s - Right (Nm) | 85.91 | 15.1 | 96.55 | 17.94 | 11 | 11.33 | 91 | 19.06 | 12 | 11.33 |
| Pamuk et al. (2020) | Isokinetic peak moment results of the knee during flexion and extension at 300°/s - Left (Nm) | 77.55 | 18.36 | 94.18 | 18.33 | 11 | 9.56 | 93 | 12.05 | 12 | 9.56 |
| Pamuk et al. (2020) | Isokinetic peak moment results of the knee during flexion and extension at 60°/s - Right (Nm) | 198.5 | 47.49 | 209.25 | 50.19 | 12 | 33.44 | 200.17 | 27.83 | 12 | 33.44 |
| Pamuk et al. (2020) | Isokinetic peak moment results of the knee during flexion and extension at 60°/s - Left (Nm) | 192.17 | 43.08 | 200.33 | 46.98 | 12 | 27.29 | 184.42 | 28.37 | 12 | 27.29 |
| Pamuk et al. (2020) | Isokinetic peak moment results of the knee during flexion and extension at 180°/s - Right (Nm) | 132.22 | 15.01 | 143.56 | 16.71 | 12 | 18.17 | 133.6 | 14.22 | 12 | 18.17 |
| Pamuk et al. (2020) | Isokinetic peak moment results of the knee during flexion and extension at 180°/s - Left (Nm) | 135.44 | 16.69 | 141.89 | 20.62 | 12 | 16.45 | 122.4 | 17.15 | 12 | 16.45 |
| Pamuk et al. (2020) | Isokinetic peak moment results of the knee during flexion and extension at 300°/s - Right | 96 | 15.54 | 95.11 | 12.54 | 12 | 11.33 | 91 | 19.06 | 12 | 11.33 |
| Pamuk et al. (2020) | Isokinetic peak moment results of the knee during flexion and extension at 300°/s - Left (Nm) | 87.67 | 20.02 | 98.89 | 18.75 | 12 | 9.56 | 93 | 12.05 | 12 | 9.56 |

**Table S5** The mean ± standard deviation (SD) of fitness variables were reported for both the plyometric jump training and control conditions in the studies included (Jumping ability).

| Reference | Test | Experimental  (pre-test) | | Experimental  (pos-test) | | n | Control  (pre-test) | | Control  (pos-test) | | n |
| --- | --- | --- | --- | --- | --- | --- | --- | --- | --- | --- | --- |
|  |  | Mean | SD | Mean | SD |  | Mean | SD | Mean | SD |  |
| Adigüzel and Günay (2016) | Vertical jumps (CMJA; cm) | 39.9 | 7 | 43.3 | 7.2 | 15 | 37.1 | 6.2 | 38.9 | 6.4 | 15 |
| Adigüzel and Günay (2016) | Vertical jumps (CMJ; cm) | 34.6 | 4.8 | 37.6 | 5.5 | 15 | 31.8 | 5.5 | 33.1 | 5.5 | 15 |
| Adigüzel and Günay (2016) | Vertical jumps (SJ; cm) | 31.9 | 3.6 | 36.1 | 4.6 | 15 | 30.1 | 6.7 | 31.7 | 6.8 | 15 |
| Amato et al. (2018) | Vertical jumps (CMJ; cm) | 29.3 | 5.5 | 31.6 | 5.7 | 12 | 27.6 | 5.9 | 26.2 | 6.2 | 11 |
| Amato et al. (2018) | Vertical jumps (SJ; cm) | 26.5 | 7.2 | 30.6 | 6.3 | 12 | 26.1 | 6.2 | 25.4 | 5.6 | 11 |
| Amato et al. (2018) | Vertical jumps (DJ; cm) | 26 | 6.5 | 27.5 | 6.2 | 12 | 24.9 | 6 | 21.9 | 5.1 | 11 |
| Andrejic (2012) | Vertical jumps (CMJA; cm) | 41.2 | 7.6 | 44.4 | 8.4 | 10 | 40.2 | 6.2 | 40.8 | 5.6 | 9 |
| Andrejic (2012) | Horizontal jumps (SLJ; cm) | 194 | 19.6 | 204 | 22.7 | 10 | 190 | 10 | 192 | 12.3 | 9 |
| Arede et al. (2019) | Vertical jumps (CMJ; cm) | 30.3 | 3.5 | 32.3 | 4.9 | 9 | 29.5 | 3.3 | 30.6 | 3.4 | 7 |
| Arede et al. (2019) | Vertical jumps (SJ; cm) | 27.2 | 2.9 | 29.4 | 3.7 | 9 | 26.9 | 3 | 27.5 | 3.2 | 7 |
| Attene et al. (2015) | Vertical jumps (CMJ; cm) | 27 | 3.6 | 30 | 3.7 | 18 | 26.1 | 3.5 | 27.3 | 4.2 | 18 |
| Attene et al. (2015) | Vertical jumps (SJ; cm) | 22.7 | 3.2 | 26.2 | 3.6 | 18 | 22.7 | 4.4 | 24.4 | 3.8 | 18 |
| Aztarain-Cardiel et al. (2024) | Vertical jumps (SJ; cm) | 30.2 | 7.1 | 32.8 | 6.8 | 10 | 32.4 | 4.2 | 32.8 | 4 | 10 |
| Aztarain-Cardiel et al. (2024) | Vertical jumps (CMJ; cm) | 33.7 | 7.7 | 35.5 | 7.7 | 10 | 36 | 4.4 | 36 | 4.3 | 10 |
| Aztarain-Cardiel et al. (2024) | Horizontal jumps (HJ; cm) | 217.6 | 31.5 | 221.1 | 25 | 10 | 219.3 | 20.6 | 221 | 26.6 | 10 |
| Aztarain-Cardiel et al. (2024) | Vertical jumps (SJ; cm) | 29 | 6.1 | 31.5 | 6.1 | 11 | 32.4 | 4.2 | 32.8 | 4 | 10 |
| Aztarain-Cardiel et al. (2024) | Vertical jumps (CMJ; cm) | 33 | 6.7 | 34.6 | 6.3 | 11 | 36 | 4.4 | 36 | 4.3 | 10 |
| Aztarain-Cardiel et al. (2024) | Horizontal jumps (HJ; cm) | 217.2 | 24.9 | 225.1 | 25 | 11 | 219.3 | 20.6 | 221 | 26.6 | 10 |
| Bouteraa et al. (2020) | Vertical jumps (CMJ; cm) | 26.8 | 3.8 | 28.8 | 3.3 | 16 | 25.2 | 2.9 | 24.4 | 3.1 | 10 |
| Bouteraa et al. (2020) | Vertical jumps (SJ; cm) | 20.4 | 3.9 | 22.5 | 3.5 | 16 | 20.4 | 2.5 | 20 | 1.9 | 10 |
| Bouteraa et al. (2020) | Vertical jumps (DJ; cm) | 24.7 | 2.9 | 28.4 | 3 | 16 | 24.8 | 1.9 | 24.6 | 2.8 | 10 |
| Bouteraa et al. (2020) | Vertical jumps (DJ; W/kg) | -0.95 | 0.08 | -0.91 | 0.05 | 16 | -1.01 | 0.08 | -0.97 | 0.06 | 10 |
| Brown et al. (1986) | Vertical jumps (CMJA; cm) | 59 | 5 | 66.3 | 5.8 | 13 | 60.4 | 6.1 | 64.1 | 4.8 | 13 |
| Brown et al. (1986) | Vertical jumps (CMJ; cm) | 49.4 | 2.9 | 54.9 | 5.6 | 13 | 51.9 | 3.9 | 54.7 | 5.6 | 13 |
| Chouhan et al. (2022) | Vertical jumps (Sargent jump test; cm) | 38 | 9.509 | 48.27 | 9.75 | 45 | 38 | 9.509 | 36.67 | 17.442 | 45 |
| Cigerci et al. (2020) | Horizontal jumps (SLJ; cm) | 194.14 | 27.79 | 209.43 | 29.27 | 10 | 183.86 | 26.02 | 186.29 | 25.3 | 10 |
| Cigerci et al. (2020) | Vertical jumps (VJ; cm) | 45.14 | 6.41 | 51.29 | 8.06 | 10 | 39.93 | 5.54 | 41.28 | 5.28 | 10 |
| Gottlieb et al. (2014) | Vertical jumps (CMJ; cm) | 41.3 | 3.9 | 42.5 | 2.7 | 9 | 40.6 | 4.8 | 42.4 | 5.8 | 10 |
| Gottlieb et al. (2014) | Horizontal jumps (6-bound jump distance; m) | 14.9 | 0.7 | 15 | 1 | 9 | 14.1 | 1.1 | 14.7 | 0.2 | 10 |
| Fontenay et al. (2013) | Vertical jumps (DJ flight time; ms) | 409 | 44 | 459 | 38 | 9 | 461 | 44 | 466 | 50 | 8 |
| Hernández et al. (2018) | Vertical jumps (CMJ; cm) | 24.1 | 5.9 | 26.9 | 5.8 | 7 | 26.2 | 7.2 | 27.5 | 7 | 6 |
| Hernández et al. (2018) | Vertical jumps (DJ; cm) | 19.6 | 6.5 | 22 | 6 | 7 | 21.5 | 4.1 | 23.5 | 3.6 | 6 |
| Hernández et al. (2018) | Vertical jumps (CMJ; cm) | 28.4 | 8.3 | 33.5 | 8.1 | 6 | 26.2 | 7.2 | 27.5 | 7 | 6 |
| Hernández et al. (2018) | Vertical jumps (DJ; cm) | 20.6 | 5.1 | 25.4 | 5.9 | 6 | 21.5 | 4.1 | 23.5 | 3.6 | 6 |
| Latorre-Román et al. (2018) | Vertical jumps (SJ; cm) | 17.5 | 4.4 | 19.5 | 4.8 | 30 | 17.2 | 3.8 | 18.4 | 4 | 28 |
| Latorre-Román et al. (2018) | Vertical jumps (CMJ; cm) | 17.85 | 4.08 | 20.36 | 4.34 | 28 | 17.47 | 4.27 | 18.51 | 4.24 | 28 |
| Latorre-Román et al. (2018) | Horizontal jumps (SLJ; cm) | 131 | 16.9 | 136 | 19.2 | 30 | 132 | 19.1 | 136 | 20 | 28 |
| Latorre-Román et al. (2018) | Vertical jumps (DJ; cm) | 15.8 | 4.1 | 17.8 | 4 | 30 | 16.4 | 3.7 | 17.4 | 4.3 | 28 |
| Matavulj et al. (2001) | Vertical jumps (CMJ; cm) | 42.1 | 4 | 46.7 | 4.3 | 11 | 38.9 | 5.9 | 38.1 | 5.8 | 6 |
| Matavulj et al. (2001) | Vertical jumps (CMJ; cm) | 37.6 | 6.5 | 43.7 | 3.6 | 11 | 38.9 | 5.9 | 38.1 | 5.8 | 6 |
| Meszler and Váczi (2019) | Vertical jumps (CMJ; cm) | 33.5 | 3.9 | 32 | 3.5 | 9 | 28.7 | 6.7 | 29.1 | 6.8 | 9 |
| Pamuk et al. (2020) | Vertical jumps (Jumping height; cm) | 56.83 | 6.91 | 58.58 | 7.01 | 12 | 58.17 | 7.64 | 62 | 6.72 | 12 |
| Pamuk et al. (2020) | Vertical jumps (Jumping height; cm) | 54.64 | 6.76 | 55.55 | 10.09 | 12 | 58.17 | 7.64 | 62 | 6.72 | 12 |
| Santos and Janeira (2008) | Vertical jumps (CMJA; cm) | 34.8 | 6.3 | 38.4 | 7.1 | 14 | 36.1 | 4.8 | 34.3 | 4.8 | 10 |
| Santos and Janeira (2008) | Vertical jumps (CMJ; cm) | 29.9 | 5.9 | 33 | 6.2 | 14 | 30.8 | 5.1 | 28.4 | 4 | 10 |
| Santos and Janeira (2008) | Vertical jumps (SJ; cm) | 24.8 | 4.2 | 28 | 4.6 | 14 | 22.7 | 4.3 | 20.7 | 3.9 | 10 |
| Santos and Janeira (2008) | Vertical jumps (DJ; cm) | 34.7 | 7.4 | 36.6 | 8.1 | 14 | 31.1 | 4.8 | 30.8 | 4.1 | 10 |
| Santos and Janeira (2009) | Vertical jumps (CMJA; cm) | 38.1 | 6.5 | 39.7 | 6.7 | 8 | 38.8 | 8.2 | 40 | 8.8 | 7 |
| Santos and Janeira (2009) | Vertical jumps (CMJ; cm) | 31.9 | 6 | 33.7 | 6.7 | 8 | 34.3 | 6.5 | 34.5 | 6.4 | 7 |
| Santos and Janeira (2009) | Vertical jumps (SJ; cm) | 27.4 | 4.1 | 26.2 | 4.9 | 8 | 28.7 | 5.4 | 26.3 | 4.2 | 7 |
| Santos and Janeira (2009) | Vertical jumps (DJ; cm) | 36.5 | 6.9 | 36.4 | 7.6 | 8 | 36.9 | 9.8 | 36.01 | 8.2 | 7 |
| Santos and Janeira (2011) | Vertical jumps (CMJ; cm) | 30.3 | 4.3 | 34.5 | 5 | 14 | 30.8 | 5.1 | 28.4 | 4 | 10 |
| Santos and Janeira (2011) | Vertical jumps (SJ; cm) | 25.2 | 3.5 | 29.2 | 4.1 | 14 | 22.7 | 4.3 | 20.7 | 3.9 | 10 |
| Santos and Janeira (2011) | Vertical jumps (DJ; cm) | 34.5 | 4.3 | 37.7 | 4.7 | 14 | 31.1 | 4.8 | 30.8 | 4.1 | 10 |
| Zribi et al. (2014) | Vertical jumps (CMJA; cm) | 33.7 | 1.4 | 37.4 | 1.3 | 25 | 33.2 | 3 | 34.1 | 1.5 | 26 |
| Zribi et al. (2014) | Vertical jumps (CMJ; cm) | 26.9 | 1.4 | 30.9 | 1.3 | 25 | 27.1 | 1.1 | 27.8 | 1.3 | 26 |
| Zribi et al. (2014) | Vertical jumps (SJ; cm) | 23.1 | 2 | 25.1 | 1.9 | 25 | 23.2 | 1.3 | 23.8 | 1.2 | 26 |
| Zribi et al. (2014) | Horizontal jumps (5-bound jump distance; m) | 9 | 0.6 | 9.5 | 0.8 | 25 | 9.1 | 0.8 | 9.3 | 0.7 | 26 |

Abbreviations CMJA, Countermovement jump with arm; CMJ, countermovement jump; SJ = squat jump; DJ, Drop jump; SLJ, Stand long jump; HJ, high jump; VJ, Vertical Jump.

**Table 6** The mean ± standard deviation (SD) of fitness variables were reported for both the plyometric jump training and control conditions in the studies included (Linear sprinting).

| Reference | Test | Experimental  (pre-test) | | Experimental  (pos-test) | | n | Control  (pre-test) | | Control  (pos-test) | | n |
| --- | --- | --- | --- | --- | --- | --- | --- | --- | --- | --- | --- |
|  |  | Mean | SD | Mean | SD |  | Mean | SD | Mean | SD |  |
| Amato et al. (2018) | Sprint 20-30m (25m sprint; s) | 5.4 | 0.4 | 5.5 | 0.4 | 12 | 5.2 | 0.4 | 11 | 0 | 11 |
| Andrejic (2012) | Sprint 20-30m (20m sprint; s) | 3.8 | 0.3 | 3.6 | 0.3 | 10 | 4.2 | 0.2 | 11 | 0 | 11 |
| Arede et al. (2019) | Sprint 0-10m (10m sprint; s) | 2.3 | 0.11 | 1.95 | 0.07 | 9 | 2.1 | 0.12 | 7 | 0.07 | 7 |
| Aztarain-Cardiel et al. (2024) | Sprint 0-10m (10m sprint; s) | 3.26 | 0.17 | 3.29 | 0.17 | 10 | 3.26 | 0.21 | 10 | 0.02 | 5 |
| Aztarain-Cardiel et al. (2024) | Sprint 0-10m (10m sprint; s) | 3.27 | 0.14 | 3.27 | 0.15 | 11 | 3.26 | 0.21 | 10 | 0.02 | 5 |
| Bouteraa et al. (2020) | Sprint 0-10m (10m sprint; s) | 1.8 | 0.1 | 1.7 | 0.1 | 16 | 1.8 | 0.1 | 10 | 0.1 | 10 |
| Bouteraa et al. (2020) | Sprint 20-30m (20m sprint; s) | 3.4 | 0.3 | 3.3 | 0.2 | 16 | 3.4 | 0.2 | 10 | 0.1 | 10 |
| Cigerci et al. (2020) | Sprint 0-10m (10m sprint;sec) | 1.94 | 0.26 | 1.72 | 0.2 | 10 | 2 | 0.24 | 10 | 0.01 | 10 |
| Cigerci et al. (2020) | Sprint 20-30m (20m sprint; s) | 3.31 | 0.16 | 3.18 | 0.14 | 10 | 3.54 | 0.33 | 10 | 0.01 | 10 |
| Gottlieb et al. (2014) | Sprint 20-30m (20m sprint; s) | 3 | 0.12 | 3 | 0.1 | 9 | 3 | 0.1 | 10 | 0 | 10 |
| Haghighi et al. (2023) | Sprint 20-30m (20m sprint; s) | 3.95 | 0.13 | 3.86 | 0.16 | 8 | 3.75 | 0.15 | 8 | 0.02 | 8 |
| Hernández et al. (2018) | Sprint 20-30m (30m sprint; s) | 5.9 | 0.5 | 5.5 | 0.6 | 7 | 5.4 | 0.4 | 6 | 0.2 | 6 |
| Hernández et al. (2018) | Sprint 20-30m (30m sprint; s) | 5.7 | 0.5 | 5.1 | 0.5 | 6 | 5.4 | 0.4 | 6 | 0.2 | 6 |
| Latorre-Román et al. (2018) | Sprint 20-30m (25m sprint; s) | 5.5 | 0.4 | 5.2 | 0.5 | 30 | 5.8 | 0.8 | 28 | 0.1 | 28 |
| Zribi et al. (2014) | Sprint 0-10m (5m sprint; m·s-1) | 3.8 | 0.3 | 4.1 | 0.3 | 25 | 3.9 | 0.2 | 26 | 0.1 | 26 |
| Zribi et al. (2014) | Sprint 20-30m (30m sprint; m·s-1) | 5.4 | 0.2 | 5.7 | 0.2 | 25 | 5.4 | 0.251 | 26 | 0.1 | 26 |

**Table S7** The mean ± standard deviation (SD) of fitness variables were reported for both the plyometric jump training and control conditions in the studies included (COD speed).

| Reference | Test | Experimental  (pre-test) | | Experimental  (pos-test) | | n | Control  (pre-test) | | Control  (pos-test) | | n |
| --- | --- | --- | --- | --- | --- | --- | --- | --- | --- | --- | --- |
|  |  | Mean | SD | Mean | SD |  | Mean | SD | Mean | SD |  |
| Amato et al. (2018) | speed-oriented COD (t-test; s) | 14.2 | 0.7 | 12.6 | 0.7 | 12 | 14 | 0.7 | 13.5 | 0.8 | 11 |
| Andrejic (2012) | strength-oriented COD (4×15m sprint; s) | 15.4 | 0.5 | 15 | 0.5 | 10 | 15.4 | 0.6 | 15.3 | 0.5 | 11 |
| Arede et al. (2019) | strength-oriented COD (Pro agility test; s) | 5.5 | 0.2 | 5.5 | 0.2 | 9 | 5.7 | 0.3 | 5.7 | 0.3 | 7 |
| Aztarain-Cardiel et al. (2024) | speed-oriented COD (V-Cut; s) | 7.05 | 0.52 | 7.05 | 0.44 | 10 | 6.96 | 0.41 | 6.99 | 0.42 | 10 |
| Aztarain-Cardiel et al. (2024) | speed-oriented COD (V-Cut; s) | 7.04 | 0.5 | 7.01 | 0.48 | 11 | 6.96 | 0.41 | 6.99 | 0.42 | 10 |
| Bouteraa et al. (2020) | strength-oriented COD (Modified Illinois test ;s) | 11.3 | 0.6 | 10.6 | 0.4 | 16 | 11.5 | 0.6 | 11.5 | 0.6 | 10 |
| Cigerci et al. (2020) | strength-oriented COD (Lane agility test; cm) | 6.21 | 0.26 | 5.89 | 0.35 | 10 | 6.35 | 0.38 | 6.31 | 0.37 | 10 |
| Gottlieb et al. (2014) | strength-oriented COD (2×5m shuttle run; s) | 1 | 0.2 | 0.9 | 0.1 | 9 | 0.9 | 0.1 | 0.8 | 0.1 | 10 |
| Fachina et al. (2017) | speed-oriented COD (t-test; s) | 11 | 1.2 | 9 | 0.2 | 18 | 11.8 | 1.2 | 11 | 0.9 | 21 |
| Haghighi et al. (2023) | strength-oriented COD (Change of Direction Sprint Ability; s) | 14.23 | 1.11 | 13.15 | 0.35 | 8 | 13.73 | 1.67 | 13.21 | 1.16 | 8 |
| Haghighi et al. (2023) | strength-oriented COD (Basketball-specific Performance; s) | 37.11 | 1.95 | 36.06 | 1.74 | 8 | 35.11 | 2.56 | 34.78 | 2.86 | 8 |
| Hernández et al. (2018) | speed-oriented COD (t-test; s) | 12.3 | 1.1 | 11 | 1.1 | 7 | 12.2 | 0.9 | 11.5 | 1.1 | 6 |
| Hernández et al. (2018) | speed-oriented COD (t-test; s) | 12.1 | 1.1 | 10.3 | 0.7 | 6 | 12.2 | 0.9 | 11.5 | 1.1 | 6 |
| Latorre-Román et al. (2018) | speed-oriented COD (t-test; s) | 15.7 | 1.4 | 14.6 | 1.3 | 30 | 16 | 1.5 | 15.9 | 1.4 | 28 |
| Meszler and Váczi (2019) | strength-oriented COD (Illinois test; s) | 16.2 | 0.8 | 17 | 1.1 | 9 | 16.9 | 0.7 | 17.3 | 0.9 | 9 |
| Meszler and Váczi (2019) | speed-oriented COD (t-test; s) | 11 | 0.5 | 11.1 | 0.6 | 9 | 11.3 | 0.51 | 11.6 | 0.8 | 9 |

**Table S8** The mean ± standard deviation (SD) of fitness variables were reported for both the plyometric jump training andcontrol conditions in the studies included (Balance).

| Reference | Test | Experimental  (pre-test) | | Experimental  (pos-test) | | n | Control  (pre-test) | | Control  (pos-test) | | n |
| --- | --- | --- | --- | --- | --- | --- | --- | --- | --- | --- | --- |
|  |  | Mean | SD | Mean | SD |  | Mean | SD | Mean | SD |  |
| Bouteraa et al. (2020) | Dynamic balance (Y-balance test; cm) | 104 | 9.7 | 114 | 10.4 | 16 | 4 | 106 | 4.2 | 10 | 4 |
| Bouteraa et al. (2020) | Static balance (Stork balance test; s) | 17.3 | 9.6 | 39.2 | 13.3 | 16 | 14.9 | 24.2 | 14.7 | 10 | 14.9 |
| Cigerci et al. (2020) | Dynamic balance (right points) | 619.19 | 48.61 | 645.95 | 75.11 | 10 | 40.19 | 657.56 | 74.18 | 10 | 40.19 |
| Cigerci et al. (2020) | Dynamic balance (Star excursion balance test;lift points) | 609.77 | 60.21 | 651.84 | 67.17 | 10 | 42.06 | 654.37 | 51.29 | 10 | 42.06 |
| McLeod et al. (2009) | Dynamic balance (Star excursion balance test, cm) | 83.1 | 6.3 | 90.6 | 6.6 | 27 | 5.8 | 86.8 | 4.2 | 23 | 5.8 |
| McLeod et al. (2009) | Dynamic balance (Balance Error Scoring System composite score; points) | 10.6 | 1.1 | 7.1 | 0.7 | 27 | 1 | 14.2 | 1.2 | 23 | 1 |
| Meszler and Váczi (2019) | Static balance (Balance with eyes open, unilateral; arbitrary units) | 74.8 | 2.2 | 75.6 | 4.3 | 9 | 7.1 | 74.1 | 5.6 | 9 | 7.1 |

**
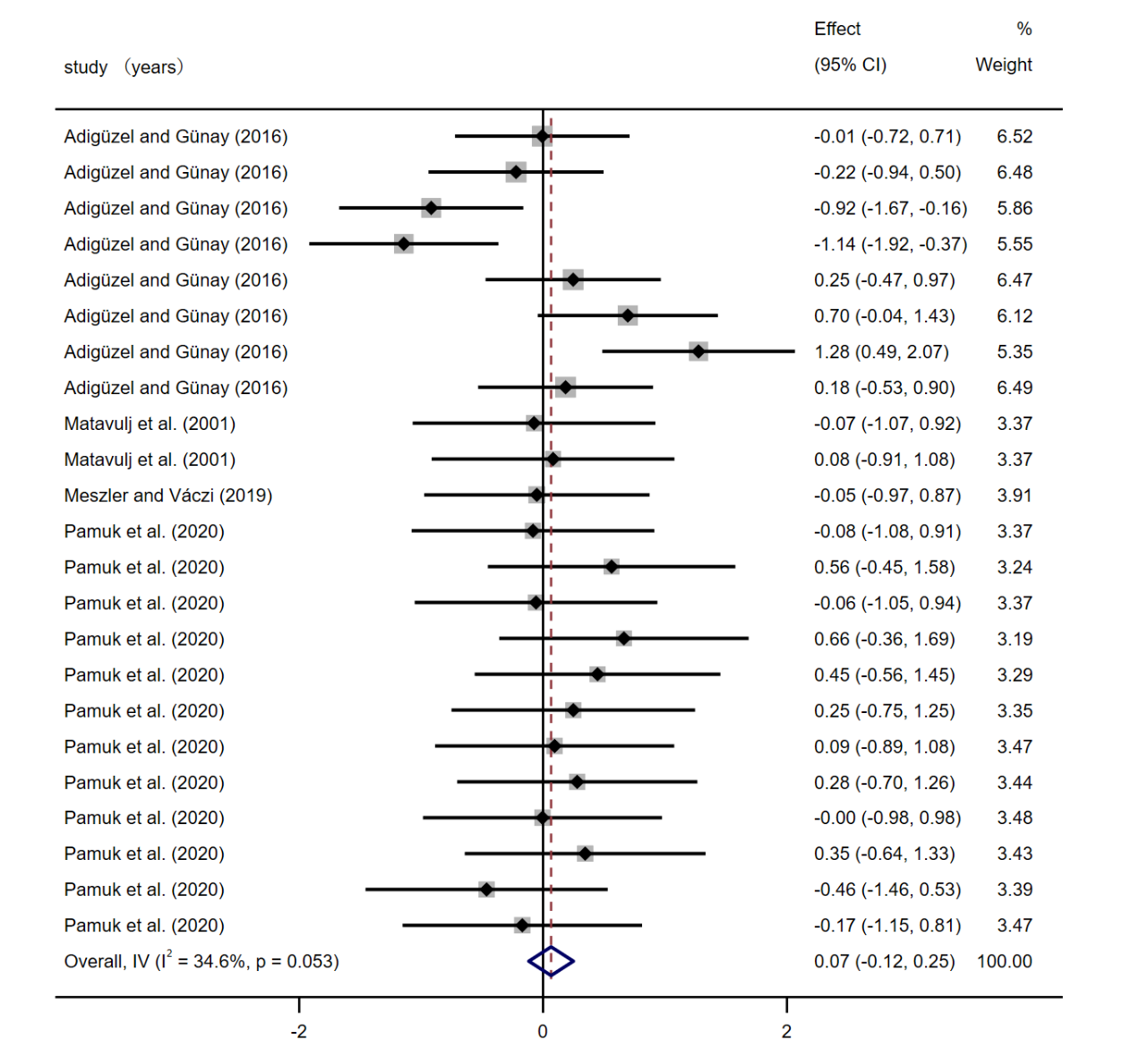
Figure S1** Results of a meta-analysis of plyometric training on lower limb strength in youth basketball players.


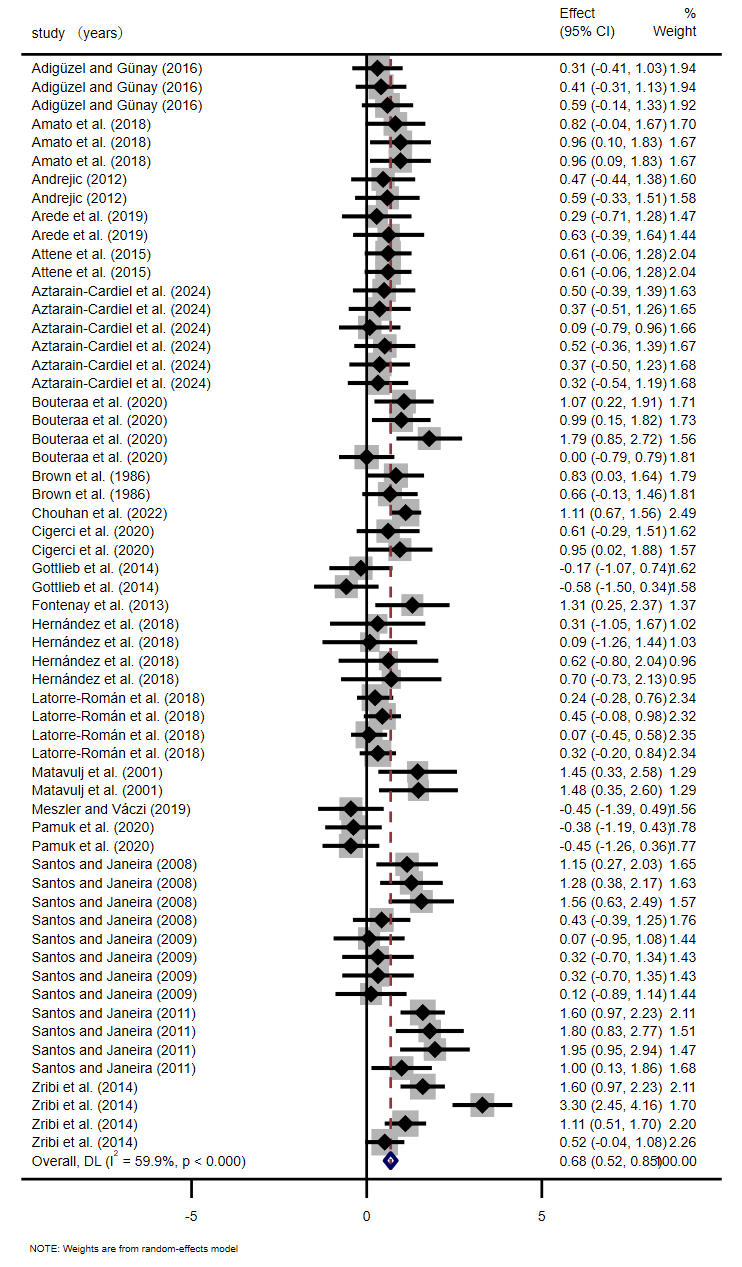
**Figure S2** Results of a meta-analysis of plyometric training on jumping ability of youth basketball players.


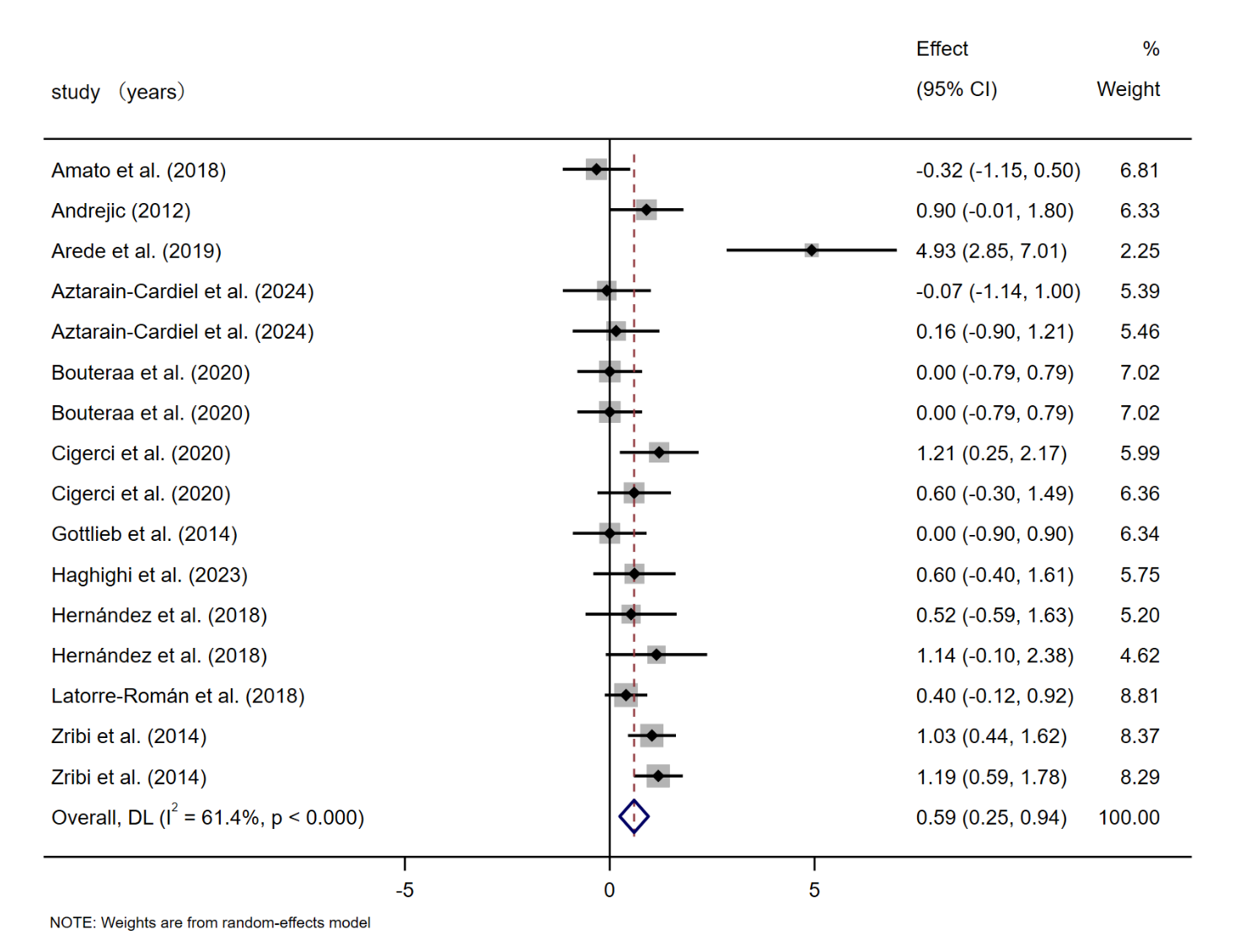
**Figure S3** Results of a meta-analysis of plyometric training on straight sprints of youth basketball players.


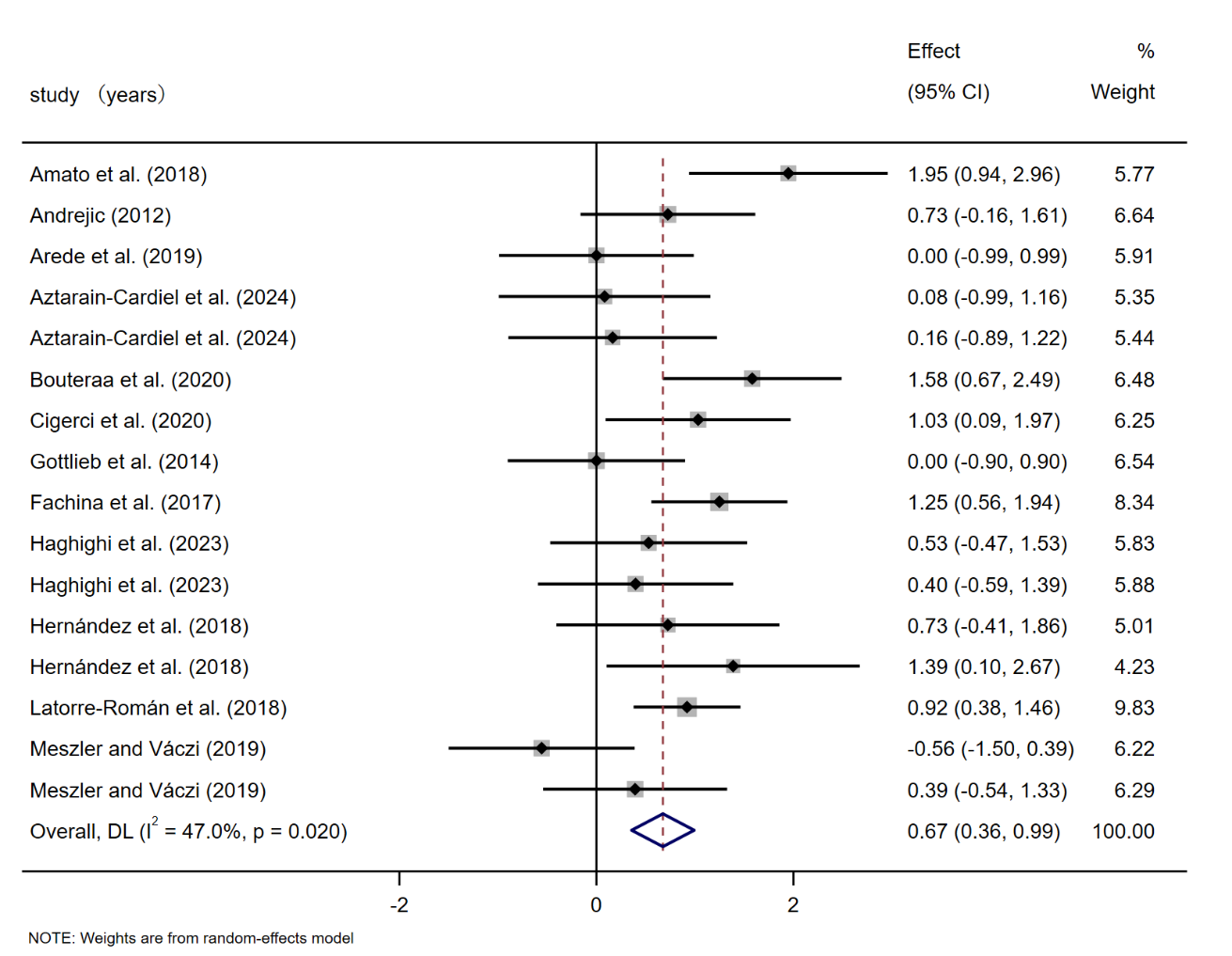
**Figure S4** Results of a meta-analysis of the effects of plyometric training on COD in youth basketball players.


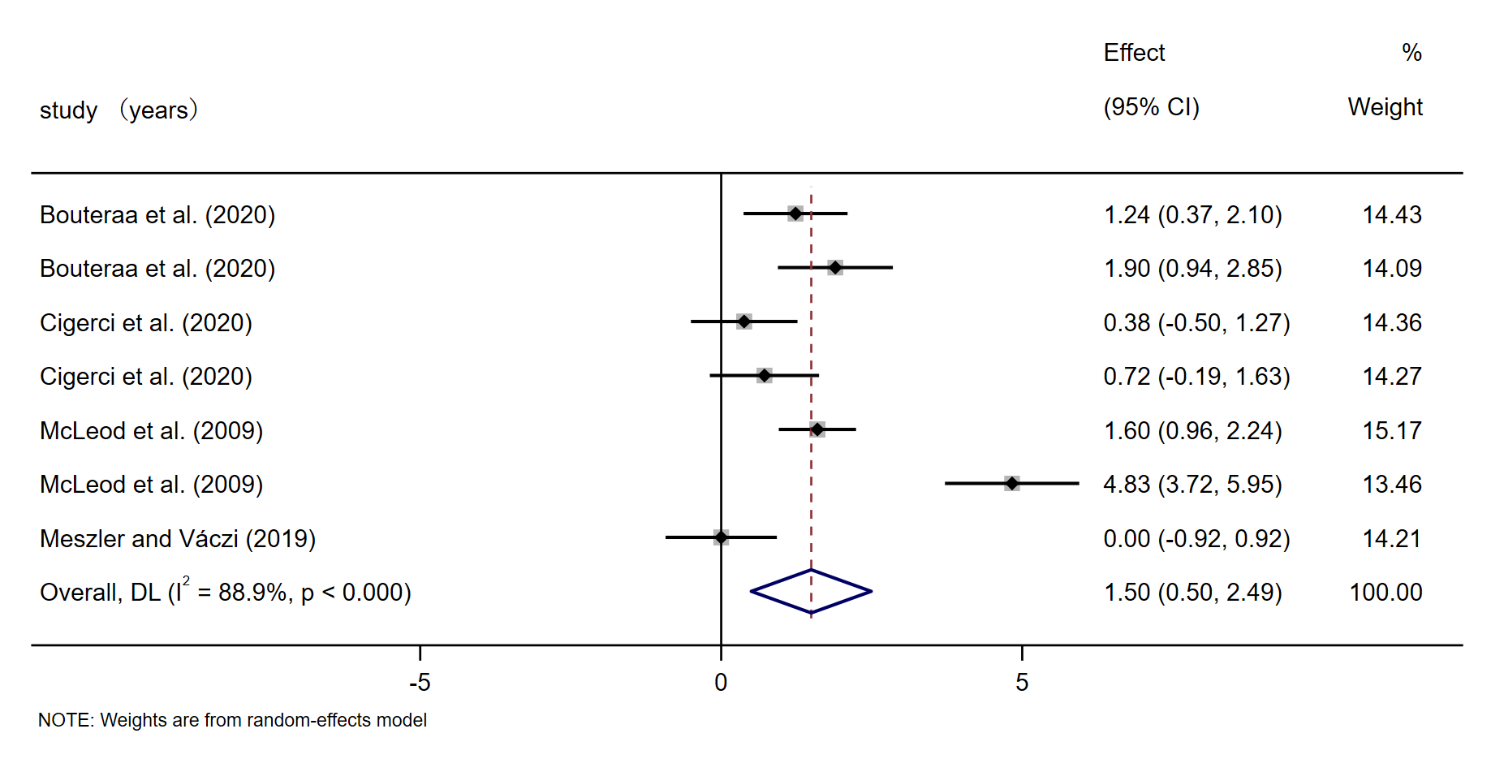
**Figure S5** Results of a meta-analysis of the effects of plyometric training on balance in youth basketball players.
